# A non-canonical GABAergic pathway to the VTA promotes unconditioned freezing

**DOI:** 10.1038/s41380-022-01765-7

**Published:** 2022-09-20

**Authors:** Loïc Broussot, Thomas Contesse, Renan Costa-Campos, Christelle Glangetas, Léa Royon, Hugo Fofo, Thomas Lorivel, François Georges, Sebastian P. Fernandez, Jacques Barik

**Affiliations:** 1 Université Côte d’Azur, Nice, France; 2 CNRS UMR7275, Institut de Pharmacologie Moléculaire & Cellulaire, Valbonne, France; 3 CNRS, IMN, UMR 5293, Université de Bordeaux, Bordeaux, France

**Keywords:** Neuroscience, Physiology

## Abstract

Freezing is a conserved defensive behaviour that constitutes a major stress-coping mechanism. Decades of research have demonstrated a role of the amygdala, periaqueductal grey and hypothalamus as core actuators of the control of fear responses, including freezing. However, the role that other modulatory sites provide to this hardwired scaffold is not known. Here, we show that freezing elicited by exposure to electrical foot shocks activates laterodorsal tegmentum (LDTg) GABAergic neurons projecting to the VTA, without altering the excitability of cholinergic and glutamatergic LDTg neurons. Selective chemogenetic silencing of this inhibitory projection, but not other LDTg neuronal subtypes, dampens freezing responses but does not prevent the formation of conditioned fear memories. Conversely, optogenetic-activation of LDTg GABA terminals within the VTA drives freezing responses and elicits bradycardia, a common hallmark of freezing. Notably, this aversive information is subsequently conveyed from the VTA to the amygdala via a discrete GABAergic pathway. Hence, we unveiled a circuit mechanism linking LDTg-VTA-amygdala regions, which holds potential translational relevance for pathological freezing states such as post-traumatic stress disorders, panic attacks and social phobias.

## Introduction

Stress is a key motor of adaptation. The stress response is mostly beneficial as it promotes survival. Whenever a living organism is presented with an environmental stressful stimulus such as a threat, it activates dedicated cerebral circuitries to select the most adaptive response among a diverse repertoire of defensive behaviours [1]. These innate and learned responses have been shaped by natural selection and conserved in both invertebrates and vertebrates. Defensive behaviours vary according to the nature of the stimulus as well as internal factors such as behavioural inhibition and anxiety traits [2, 3]. In terms of behavioural outputs, defensive behaviours range from passive strategies such as freezing to active fight-or-flight responses, and the switch between these passive/active modes is essential for behavioural flexibility [1, 4, 5].

Freezing is a universal fear response characterised by a total lack of movement, aside breathing, due to a tense body posture when a threat is encountered. Freezing is cardinal in stress-coping processes as it corresponds to a state of hypervigilance, enabling decision-making and consequently building the most pertinent behavioural strategy. Although freezing has relevance for the etiology of threat-related disorder such as post-traumatic stress disorders (PTSD), panic attacks and social phobias [6–9], the underpinning neuronal circuits and cellular substrates are far from being understood. A large body of evidence indicates that cerebral structures such as the periaqueductal grey (PAG), the hypothalamus, or the amygdaloid complex play a major role in the detection, integration and response to unconditioned and conditioned threats in both rodents and humans [2, 10, 11]. Indeed, decades of work using different approaches ranging from lesions, pharmacological interventions and electrical stimulations to more recent opto- and pharmacogenetic tools positioned the amygdala as a core unit in this hierarchical network of fear defensive system [11]. The lateral amygdala computes information from sensory and associative inputs from cortices and thalamic nuclei that is conveyed to the central amygdalar output nucleus [2, 11]. This latter influences the activity of PAG and hypothalamic pathways to subsequently influence the activity of the medulla and pons resulting in changes in viscera function, muscle contraction and pain sensitivity [12]. Yet, in light of the impact of stress on the brain, stress-coping is likely to recruit key modulatory brain networks, which could shape freezing responses. Uncovering these pathways is an important step to fully understand the etiology of fear responses such as freezing, and to build a comprehensive functional map of these underlying interconnected subcortical and cortical regions. In humans, this distributed defensive network can suffer unduly activation and persistent deregulations sustained by epigenetic and synaptic changes, which clinically manifest as states of intense distress, peri-traumatic reactions and PTSD [13].

The laterodorsal tegmental nucleus (LDTg) is interconnected with limbic regions and responds to somatosensory, visual and auditory stimuli [14]. Although primarily studied for its role in reward-oriented behaviours [15–17] and paradoxical sleep [18, 19], recent work indicates that the LDTg can convey stress-related information [20, 21]. The LDTg constitutes a strong modulator of the motivational balance regulating appetitive and negatively-valenced behaviours. As part of the reticular formation, the LDTg contributes also to behavioural arousal thus facilitating sensory integration [22, 23], which is a key component affecting fear responses. Hence, we hypothesised that the LDTg could contribute to defensive behaviour such as freezing responses. To test this, we combined electrophysiological in vivo and ex vivo recordings and behavioural analyses to assess electric shocks-induced freezing. To causally link the LDTg to freezing responses, we employed pharmacogenetic and optogenetic approaches in virally-tagged brain circuits. We uncover a non-canonical pathway implicating LDTg GABAergic projection to the ventral tegmental area (VTA), which subsequently impact amygdalar neurons to regulate unconditioned freezing responses.

## Material and methods

### Animals

All procedures were in accordance with the recommendations of the European Commission (2010/63/EU) for care and use of laboratory animals and approved by the French National Ethical Committee (#9185-2017020911476246 and #16459-2018061116303066). We used male C57BL/6J mice (Janvier Labs, France), vGAT-CRE mice (The Jackson Laboratory, stock number: 028862), vGluT2-CRE mice [24], and ChAT-CRE mice (The Jackson Laboratory, stock number: 006410). Transgenic mice were heterozygous and backcrossed on a C57BL/6J background. Mice were housed 4–5 mice per cage on a 12 h/12 h light/dark cycle with lights on from 8:00 a.m to 8:00 p.m. Mice had free access to food and water ad libitum. Enriched housing consisted of a chewing block of wood, a plastic igloo and cotton to facilitate nesting. All mice used in behavioural experiments were handled daily for 1 week before each test to limit any stress induced by intraperitoneal injections and connections to laser cables for in vivo optogenetic experiment. All tests were performed on adult mice that were at least 2 months old and littermates were used as controls. All the experiments were performed in accordance to the ARRIVE guidelines.

### Reagents and drug administration

Clozapine-N-Oxide (CNO) was purchased from Enzo Life (France), Ketamine and Xylazine from Centravet (France), and picrotoxin from Sigma-Aldrich (France). All drugs for in vivo administration were diluted in saline solution 0.9% NaCl. Mice received either saline (10 mL/kg) or CNO (1 mg/kg) as previously described in ref. [20].

### Viral tools

Viruses were purchased from the following facilities: Addgene (plasmid #50475 AAV8-hSyn-hM4D(Gi)-mCherry, plasmid #44362 AAV8-hSyn-DIO-hM4D(Gi)-mCherry, plasmid #44361 AAV8-hSyn-DIO-hM3D(Gq)-mCherry, plasmid #20298 AAV5-EF1a-double floxed-hChR2(H134R)-EYFP-WPRE-HGHpA, plasmid #20297 AAV5-EF1a-double floxed-hChR2(H134R)-mCherry-WPRE-HGHpA) and Plateforme de vectorologie de Montpellier (Canine Adenovirus type 2, CAV-2-Cre). For modulation in a projection- and cell-type specific manner we used viruses from Zurich vector core facility (identifier: v190-8 AAV-8/2-hSyn1-dFRT-hM4D(Gi)-mCherry(rev)-dFRT-WPRE-hGHp(A), identifier:v171-retrograde ssAAV-retro/2-hSyn1-chI-dlox-EGFP_2A_FLPo(rev)-dlox-WPRE-SV40p(A)), Titre for AAVs and CAV-2-Cre were ≥10^12^ ppl/mL and ≥10^13^ ppl/mL respectively.

### Stereotaxic injections

Stereotaxic injections were performed using a stereotaxic frame (Kopf Instruments). General anaesthesia was achieved using a mix of ketamine (150 mg/kg) and xylazine (10 mg/kg). All viruses were injected bilaterally at a rate of 100 nL/min for a final volume of 200 nL per site (except for VTA for which we injected 300 nL). Mice were 5–6 weeks old at the time of surgery and were given at least a 3-week-recovery period for pharmacogenetic experiments and 6 weeks for optogenetic tests to allow sufficient viral expression. Stereotaxic coordinates (antero-posterior: AP; Mediolateral: ML; dorsoventral: DV) were based on the Paxinos atlas of the adult mouse brain [25], and adapted as we performed surgeries on young mice. They are given here in millimetres (mm) from bregma for AP and ML coordinates, DV is taken from skull at the site of injection. Coordinates were as followed: LDTg: AP −4.70, ML ± 0.50, DV −3.60; VTA: AP −2.80, ML ± 0.60, DV −4.70; CeA: AP −0.75, ML ± 3.00, DV −5.05; vlPAG: AP −4.00, ML ± 0.60, DV −2.50; BLA: AP −1.30, ML ± 3.20, DV −4.60; LS: AP + 1.0, ML ± 0.2, DV −3.35.

To silence LDTg neurons, an AAV8-hSyn-hM4D(Gi)mCherry was injected bilaterally in the LDTg of C57Bl6J mice.

To specifically silence LDTg cholinergic, glutamatergic and GABAergic neurons, an AAV8-hSyn-DIO-hM4D(Gi)mCherry was injected bilaterally in the LDTg of ChATCre, vGluT2Cre and vGATCre mice respectively.

For projection-specific silencing, wild-type mice were bilaterally injected with CAV-2-Cre in the periaqueductal grey (PAG), central amygdala (CeA) or VTA and with a hSyn-DIO-hM4D(Gi)-mCherry in the LDTg (hereafter named LDTg^hM4→PAG^, LDTg^hM4→CeA^, LDTg^hM4→VTA^ mice). For selective manipulation of VTAg^→BLA^ projections we injected bilaterally a CAV-2-Cre in the BLA and a hSyn-DIO-hM4D(Gi)-mCherry in the VTA.

To selectively activate LDTg to VTA projections we injected a CAV-2-Cre in the VTA and a hSyn-DIO-hM3D(Gq)-mCherry in the LDTg of wild-type mice (hereafter named LDTg^hM3→VTA^).

For projection- and neurotransmitter-specific manipulation, vGATCre mice were bilaterally injected with a retrograde AAV-retro/2-hSyn1-chI-dlox-EGFP_2A_FLPo(rev)-dlox-WPRE-SV40p(A) in the VTA and an AAV-8/2-hSyn1-dFRT-hM4D(Gi)-mCherry(rev)-dFRT-WPRE-hGHp(A) in the LDTg (hereafter named GAT^hM4; LDTg→VTA^).

### In vivo optogenetic manipulation

Optical fibres (200 μm core, 0.39 NA, made following the protocol of [26] were implanted bilaterally into the VTA with a 15° angle (AP: −2.8 mm; ML: −1.5 mm; DV: −4.2 mm), 4–5 weeks after viral injection. Fibre optics were connected by a mating sleeve (ThorLabs, ADAL1-5) to patch-cords (Doric Lenses, MFP_200/240/900-0.22_1m_FC-ZF1.25(F)) to a fibre optic rotary joint (Doric Lenses FRJ_1x2i_FC-2FC_0.22) itself connected to a laser source (ThorLabs S1FC473MM). Mice were habituated to fibre optic connection for 30 min per day during the week preceding behavioural testing. On the day of experiment, mice performed behavioural tests after 10 min [21] habituation following connection. The power of the blue (470 nm) laser was 10 mW mm^−2^ as measured at the tip of the optic fibre. Light stimulation was delivered at 50 Hz in pulses of 20 ms for periods of 1 min. Control group mice underwent the same procedure and received the same intensity of laser stimulation. Mice with misplaced viral injections or fibre optic implantations were excluded. The stimulation parameters were based on both the ex vivo electrophysiological recordings performed in our laboratory and also published literature [21].

For the conditioned place aversion test, we used two distinct chambers (with different visual and tactile cues) connected by a neutral compartment. On day 1, control and vGAT-Cre ChR2 mice freely explored the apparatus during 20 min. From day 2 to 4, mice were randomly assigned to a paired chamber (pairing was counterbalanced) and received light stimulation (50 Hz frequency, 20 ms duration, delivered at 5 min duration with 5 min intervals). Two conditioning sessions of 20 min were conducted each day, so that mice received light stimulation in the paired chamber and no light stimulation in the unpaired chamber (am and pm sessions counterbalanced). On day 5, mice freely explored the apparatus in absence of stimulation. Results are expressed as different between the time spent (s) in paired chamber between day 5 (test) and day 1 (pre-test).

### Behavioural testing

In all behavioural tests, unless otherwise stated, mice were housed in a habituation room adjacent to the experimental room at least 1 h before the beginning of the test. Unless otherwise stated, saline or CNO were injected 30 min before behavioural testing. After each test, mice were temporarily housed in a new cage to avoid social interaction with untested mice until they were all tested and finally returned to their home cages.

### Freezing paradigm

To measure electric shock-induced freezing, each mouse was placed individually in a soundproof test chamber containing a floor made of a grid with 27 stainless-steel rods (diameter 4 mm) spaced 1 cm apart and connected to a generator to allow shock delivery (Shocker LE 100-26 Panlab Harvard Apparatus Bioseb). Mice were left to freely explore the apparatus for 3 min and then received three consecutive electrical foot shocks (intensity: 0.7 mA, duration: 2 s) with a 58 s interval between each shock. Mice remained in the test chamber 1 min after the last foot shock. Activity levels of mice were recorded through a high-precision sensor plate placed beneath the floor grid (Load cell coupler LE 111 Panlab Harvard Apparatus Bioseb) to assess the variations of weight induced by the movements of the mice. Each test was also recorded using a video camera (Samsung SDP-860) placed above the apparatus. Freezing was defined as total lack of movement aside from breathing for a cumulative duration of at least 2 s. An experimenter blind to the experimental conditions manually scored the freezing behaviour of each mouse using the video recordings.

The subthreshold version of the freezing paradigm used with the activatory hM3 DREADD system was run over 2 days. On the first day, mice were placed in the test chamber as described above and left to explore freely for 5 min. On the second day, mice received a saline or CNO injection and immediately introduced in the test chamber. Mice were left 1 min to explore freely and then received two electrical foot shocks (intensity: 0.7 mA, duration: 2 s; 58 s interval). Mice were then left undisturbed for 15 min and freezing behaviour was assessed using the same method as described above during the last 5 min of the test.

All mice employed in the freezing and its subthreshold version were not re-used in other behavioural tests.

For in vivo optogenetic manipulations, mice were put in the same test box and left free to explore the apparatus for 5 min (habituation, OFF). Then, they received intracerebral illumination for 1 min (ON), and then 1 min without illumination (OFF). No electrical shocks were delivered. To test for aversive memory formation, mice were re-exposed 24 h after to the same context 24 h. The video camera was placed on the side of the test chamber for further behavioural analyses.

### O-maze

The O-maze test was used to measure anxiety levels. Each mouse was placed in a circular maze consisting of two open arms and two closed arms alternating in quadrants (width of walking lane: 5 cm, total diameter: 55 cm, height of the walls: 12 cm, elevation above the floor: 60 cm) with an ambient light of 200 lux in the open arms. Mice movements were recorded during 5 min using a video camera placed above the maze. An experimenter blind to the experimental groups scored the time spent in the open arms.

### Open-field

The Open-field test was used to measure locomotor activity. Each mouse was placed in a 40 × 40 cm open field with an ambient light of 200 lux for 5 min and left to explore freely. Mice movements were recorded using a video camera placed above the apparatus. Distanced travel was automatically analysed using the software AnyMaze (Stoelting, France).

### Hargreaves test

The Hargreaves test was used to measure pain sensitivity. Each mouse was placed in a small transparent plastic compartment in a room lit by red light for 30 min of habituation. Then, a radiating infrared source (intensity: 190 ± 1 mW cm²) was placed under the rear paw of the mice and the latency to withdraw the paw was measured. Each day, two measures were taken for each rear paw with at least 1 min between each measure and the mean latency was calculated. The test was repeated during 3 days with 1 day of interval between each trial. Only the results of the last two trials were analysed by a blind experimenter. Of note, the mice which underwent the Hargreaves test were first tested in the O-maze and then Open-field with 4 days interval between each test to avoid potential effect of pre-CNO exposure.

### Ex vivo patch-clamp recording

Mice were anesthetised (ketamine 150 mg/kg, xylazine 10 mg/kg) and transcardiacally perfused with aCSF for slice preparation. For LDTg recordings, 250 µm coronal slices were obtained in bubbled ice-cold (95% O2/5% CO2) aCSF containing (in mM): KCl 2.5, NaH_2_PO_4_ 1.25, MgSO_4_ 10, CaCl_2_ 2.5, glucose 11, sucrose 234, and NaHCO_3_ 26. Slices were then incubating in aCSF containing (in mM): NaCl 119, KCl 2.5, NaHPO_4_ 1.25, MgSO_4_ 1.3, CaCl_2_ 2.5, NaHCO_3_ 26, and glucose 11 at 37 °C for 15 min, and then kept at room temperature. Slices were transferred and kept at 32–34 °C in a recording chamber superfused with 2.5 mL/min aCSF. Visualised whole-cell voltage-clamp or current-clamp recording techniques were used to measure synaptic responses or excitability respectively, using an upright microscope (Olympus France). Current-clamp experiments were obtained using a Multiclamp 700B (Molecular Devices, Sunnyvale, CA). Signals were collected and stored using a Digidata 1440 A converter and pCLAMP 10.2 software (Molecular Devices, CA). In all cases, access resistance was monitored by a step of −10 mV (0.1 Hz) and experiments were discarded if the resistance increased more than 20%. Internal solution contained (in mM): K-D-gluconate 135, NaCl 5, MgCl_2_, HEPES 10, EGTA 0.5, MgATP 2, and NaGTP 0.4. Depolarising (0–300 pA) or hyperpolarizing (0–450 pA) 800 ms current steps were used to assess excitability and membrane properties of LDTg and VTA neurons. For VTA recordings, 250 µm horizontal slices were obtained as before, and incubated in aCSF for 1 h at 37 °C before recording started.

To probe for functional synaptic connections within the VTA, we injected vGATCre mice with an AAV-DIO-ChR2-YFP in the LDTg and an AAV-hSyn-DIO-mCherry in the VTA. This allows us to identify VTA GABAergic neurons expressing mCherry while opto-genetically activating GABAergic LDTg terminals. In another batch of mice, we performed the same injections in the LDTg and putative VTA DA neurons were identified based on classical criteria and as previously done [20, 27]. To unambiguously identify VTA glutamatergic neurons, we injected vGluT2Cre mice with AAV-hSyn-DIO-mCherry in the VTA and a non cre-dependent AAV-hSyn-ChR2-YFP in the LDTg. We pharmacologically isolated the GABAergic component of the optogenetic stimulation using glutamatergic receptor antagonists (AP5 50 μM and DNQX 10 μM) and cholinergic receptor antagonists (mecamylamine 10 μM and atropine 1 μM). Last, to record VTA^→BLA^ neurons, we injected vGATCre mice with an AAV-DIO-ChR2-YFP in the LDTg and a retro AAV-hSyn-tdTomato in the BLA. VTA horizontal sections were prepared as previously done [20]. Whole-cell voltage-clamp recordings of fluorescent-tagged neurons in VTA were performed using the same internal solution as before with Vm = −40 mV in the presence of DNQX to block AMPA currents. Photocurrents were induced by optical illumination (0.1 Hz) with 5 ms blue light pulses delivered by a LED through the objective light path. Twenty sweeps were averaged offline, and peak amplitude was measured to assess light-evoked current size. GABA-A receptors mediated currents were blocked using picrotoxin (Sigma, France) bath applied to a final concentration of 50 µM.

In all cases, offline analysis was performed using Clampfit 10.2 (Axon Instruments, U.S.A.) and Prism (Graphpad, U.S.A.).

### Immunohistofluorescence

To achieve fixation of the brains, mice were anesthetisized using a mix of ketamine (150 mg/kg) and xylazine (10 mg/kg) then underwent transcardial perfusion with cold phosphate buffer (PB 0.1 M Na_2_HPO_4_/NaH_2_PO_4_, pH 7.4) followed by paraformaldehyde (PFA 4%, diluted in PB 0.1 M). Brains were left at 4 °C in PFA 4% overnight, then cut into 40 µm free-floating slices. Sections were used to assess the correct location of stereotaxic viral injection for each animal and cannula implantation for in vivo optogenetic experiments.

For cFos labelling, brain sections containing the nucleus accumbens, amygdala or lateral septum were incubated (30 min) in PBS-BT (PBS 0.5% BSA, 0.1% Triton X-100) with 10% normal goat serum (NGS). Sections were then incubated (4 °C) in PBS-BT, 1% NGS, with primary anti-cFos (1:1000, Abcam anti-rabbit1:1000, Cat Ab190289) for 36 h. Sections were rinsed in PBS and incubated (2 h) in goat anti-rabbit Alexa488 secondary antibody (1:1000, Vector Laboratories, Burlingame, CA; Cat goat anti-rabbit Alexa488 secondary antibody (1:1000, Vector Laboratories, Burlingame, CA; Cat. DI-1488-1.5) in PBS-BT, 1% NGS. Sections were rinsed with PBS and incubated 5 min with DAPI before mounting with Mowiol. Images for quantification of cFos-positive cells were acquired using a TCS SP5 confocal microscope (Leica Microsystems). An experimenter blind to treatment counted manually using ImageJ. Counting was done on two adjacent brain sections and the two hemispheres for each region analyzed. A mean value was averaged for each mouse and plotted for each experimental condition (i.e. Control or ChR2) as cFos+ cells/mm^2^.

### In vivo single-unit neuron recordings

We performed as previously published [28, 29]. Briefly, stereotaxic surgeries for electrophysiology experiments were performed under 1.0–1.5% isoflurane (in 50% air/50% O_2_; 1 L/min) anaesthesia. A glass micropipette filled with 2% pontamine sky blue solution in 0.5% sodium acetate was lowered into the VTA (−3.16 mm/bregma, 0.5 mm/midline, 3.5–4.5 mm/brain surface) [25]. Extracellular potentials were recorded with an Axoclamp-2B amplifier and filter (300 Hz/0.5 Hz) [30]. Spikes were collected online (CED 1401, SPIKE 2; Cambridge Electronic Design; UK). VTA dopamine neurons were identified according to well-established electrophysiological features [31, 32], which included the following (i) an action potential with ≥1.1 ms (measured from the start of action potential to the negative trough), (ii) spontaneous firing rate (≤10 Hz) (iii) single and burst spontaneous firing patterns composed by 2 to 10 spikes in vivo. The onset of a burst was defined with an interspike interval lower than 80 msec and the end of the burst with an interspike interval higher than 160 ms [33]. Putative VTA GABA neurons were identified according to well-established electrophysiological criteria (i) an action potential <1 ms; (ii) VTA boundary was defined 100 µm dorsal to the first VTA DA neuron [33–36]. Several parameters for VTA dopamine neuron firing and bursting were analyzed over a 100 s period: the cumulative probability distribution of the firing rate and the bar graphs with the mean ± SEM, the bursting rate, the percentage of spikes in burst (% SIB). Firing frequency of GABA neurons was analyzed over a 100 s period. Results are expressed as mean ± SEM.

### Heart-rate recordings

Heart rate was recorded using the MouseOx Pulse non-invasive oximeter (Starr Life Sciences). Briefly, mice were shaved around the neck area 24 h before recordings. On the recording day, individual mice were anaesthetised and maintained under 1.0–1.5% isoflurane (in 50% air/50% O_2_; 1 L/min). The neck collar and system were set up according to manufacturer instructions, and optic fibres were connected to the head to stimulate LDTg GABA terminals in the VTA. After obtaining a baseline of 10 min, 3 photostimulation protocols were delivered at 50 Hz for 1 min, separated by 2 min. Heart-rate was acquired at 5 Hz sampling rate, and analyzed using Excel. Heart-rate deviations from baseline were calculated by building a z-score of the whole trace using the mean and SD. Data are expressed as mean ± SEM.

### Data analysis

Data were analysed using Prism (GraphPad, U.S.A.). Normality of the distribution was first tested using a Kolmogorov–Smirnov test. Depending of the number of groups and the results of the normality test, groups were then compared using a Student *t* test, *U* of Mann–Whitney, or two-way analyses of variance followed by post hoc Sidak’s test. Data in the figures are presented as mean ± SEM. Statistical significance was conventionally established at *p < 0.05; **p < 0.01; ***p < 0.001.

## Results

### LDTg inputs to the VTA bidirectionally control freezing

**Fig. 1:**
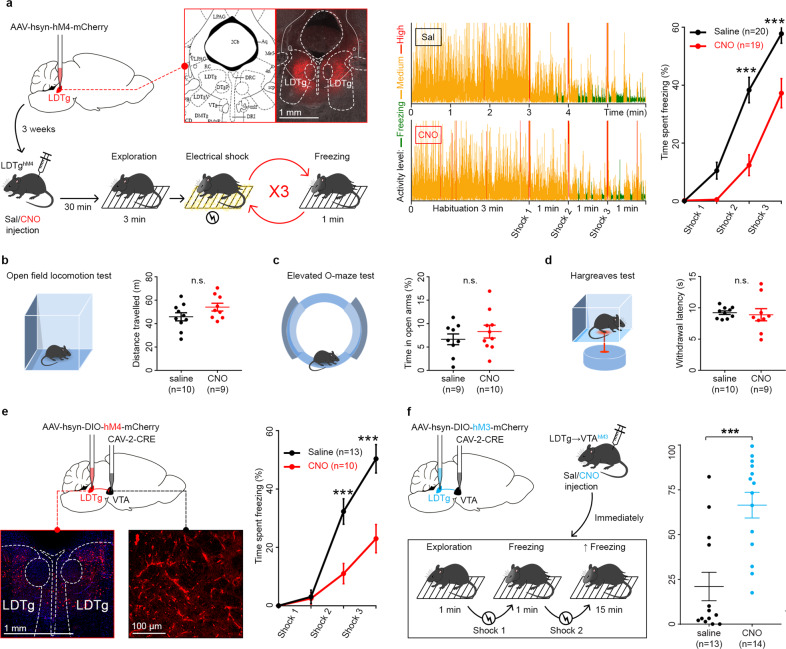
LDTg inputs to the VTA bidirectionally control freezing. **a** Left panel: wild-type mice were injected with AAV-hsyn-hM4-mCherry in the LDTg. Microscopy image, opposed to coronal LDTg anatomy from reference [25], shows red fluorescence at correct injection site. Three weeks later, LDTg^hM4^ mice received saline or CNO injection 30 min prior to exposure to three electrical foot shocks. Middle panel: representative traces showing activity of mice during the test. Right panel: freezing responses to electrical foot shocks are decreased in mice treated with CNO compared to saline. Points represent mean ± S.E.M. percentage of time spent freezing during the following time intervals: 0–3 min, 3–4 min, 4–5 min, and 5–6 min. Interaction treatment × shocks *F* (3, 111) = 10.12; repeated measures two-way ANOVA followed by Sidak’s comparison test, ***P < 0.001). **b** Locomotor activity in open-field was not affected by LDTg inhibition (*P* = 0.1072, *t* test). **c** Anxiety levels were not affected in O-maze test (*P* = 0.3741, *t* test). **d** Pain sensitivity was not affected in Hargreaves test (*P* = 0.7339, *t* test). **e** Wild-type mice were injected with AAV-hsyn-DIO-hM4-mCherry in LDTg and CAV-2-CRE in VTA. Chemogenetic silencing of LDTg projections to VTA reduces freezing. Interaction treatment × shocks *F* (3, 63) = 11.56; repeated measures two-way ANOVA followed by Sidak’s comparison test, ***P < 0.001). **f** Hyperactivation of LDTg projections to VTA increases freezing. Same injections as “e” except hM4 (Gi-coupled, inhibitory) was replaced with hM3 (Gq-coupled, excitatory). Mice received only two shocks and their freezing response was measured 10 min after the last shock delivery, for 5 min. Percentage of time mice spent freezing (***P < 0.001, Mann–Whitney test).

To assess the involvement of the LDTg in processing freezing, we used a chemogenetic approach to remotely silence LDTg neurons prior administration of electrical foot shocks. Wild-type C57BL/6J mice were injected into the LDTg with inhibitory DREADD (AAV-hSyn-hM4D-mCherry), hereafter referred to as LDTg^hM4^ mice (Fig. 1a, left panel). As depicted in the activity chart, foot shock delivery produced a decrease in locomotor activity and an increased time spent in freezing postures (Fig. 1a, middle panel). Quantification of freezing in saline-treated mice showed a gradual increase in this behaviour after each foot shock. In contrast, silencing of LDTg neurons engendered a pronounced and significant downward shift of the freezing response curve (Fig. 1a, right panel). This reduction of freezing was not caused by changes in locomotor activity, anxiety levels or pain sensitivity since saline- and CNO-treated LDTg^hM4^ mice showed similar responses in the open-field, elevated O-maze and Hargreaves test (Fig. 1b, c, d respectively). Importantly, reduced freezing did not impair aversive memory formation since re-exposure to the same context 24 h after (Day 2) produced comparable conditioned-freezing responses in both groups (Fig. S1). These results demonstrate that activity in LDTg neurons is necessary for fear freezing manifestation but not memory formation of an aversive event.

To unravel the circuitry that links LDTg and freezing, we carried projection-specific chemogenetic manipulations of LDTg projections to the ventrolateral periaqueductal grey (vlPAG) and the central amygdala (CeA), two regions classically linked to the control of freezing responses [11, 37]. We therefore injected a retrograde CAV-2-Cre in either the vlPAG or the CeA and a Cre-dependent inhibitory DREADD (AAV-hSyn-DIO-hM4D-mCherry) in the LDTg to independently manipulate LDTg^→vlPAG^ or LDTg^→CeA^ projections. We validated this approach by immunohistofluorescence as we observed mCherry-positive neurons in the LDTg and red fibres within the CeA and vlPAG (Fig. S2a, b respectively). Silencing LDTg^→vlPAG^ or LDTg^→CeA^ projections did not alter the freezing response that was similar in mice receiving either saline or CNO (Fig. S2a, b respectively). Next, given the rising interest of the VTA in aversion processing [38, 39], and the dense projections arising from the LDTg [40–43], we hypothesised that this pathway could be involved in freezing behaviours. Indeed, silencing LDTg^→VTA^ projections triggered a significant downward shift of the freezing response (Fig. 1e), resembling results obtained with full LDTg silencing (Fig. 1a). Given this result, we hypothesised that activating this pathway may exacerbate fear responses to a mild aversive challenge. Thus, we used excitatory DREADD (AAV-hSyn-DIO-hM3D-mCherry) in order to stimulate LDTg^→VTA^ projections while exposing mice to only two foot shocks. CNO-injected mice exhibited a strong freezing response indicating that a mild stress challenge primed LDTg^→VTA^ projections (Fig. 1f). These results point to the specificity of discrete LDTg circuits to the aversive processing of electric shocks.

### LDTg GABAergic neurons are required for freezing

**Fig. 2:**
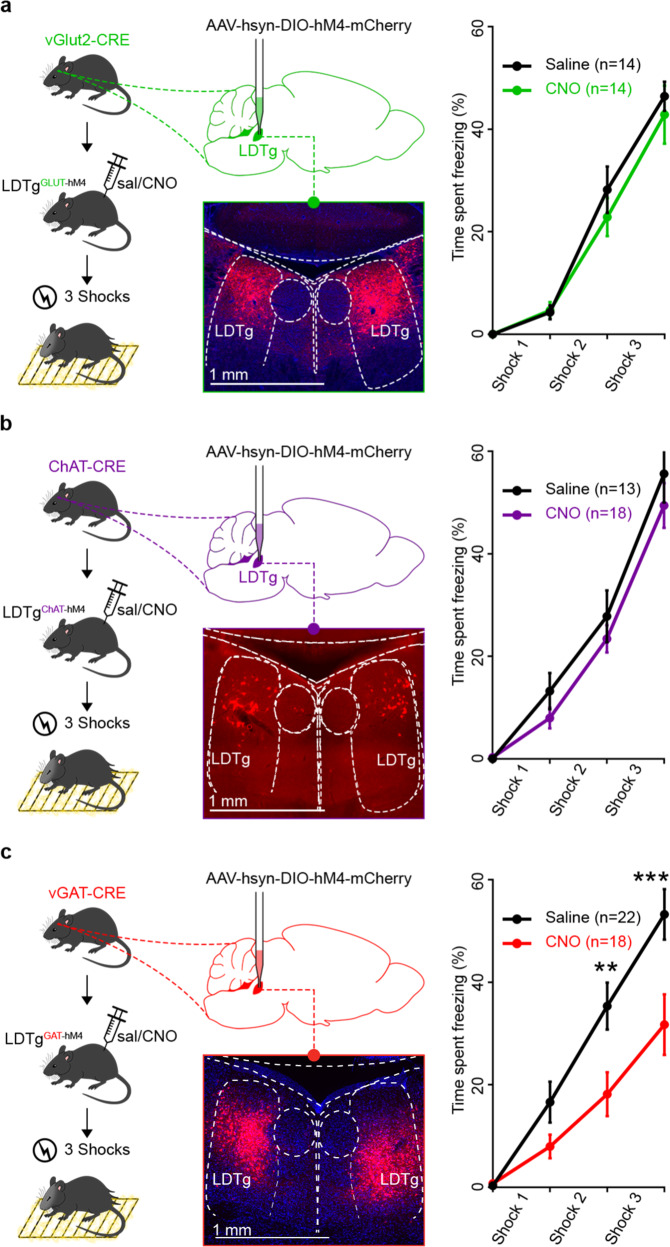
LDTg GABAergic neurons but not cholinergic or glutamatergic cells control freezing. **a** Silencing glutamatergic LDTg neurons does not alter freezing. vGluT2-cre mice received injection of AAV-hsyn-DIO-hM4-mCherry in LDTg then underwent freezing paradigm; microscopy image shows mCherry expression at injection site. Percentage of time mice spent freezing on each interval, same as Fig. 1 (interaction treatment × shocks *F* (3, 78) = 0.4895; repeated measures two-way ANOVA followed by Sidak’s comparison test, *P* = 0.6906). **b** Silencing cholinergic LDTg neurons does not alter freezing. Same as above except mice were ChAT-cre (interaction treatment × shocks *F* (3, 87) = 0.4895; repeated measures two-way ANOVA followed by Sidak’s comparison test, *P* = 0.6117). **c** Silencing GABAergic LDTg neurons reduces freezing. Same as above except mice were vGAT-cre (interaction treatment × shocks *F* (3, 114) = 5.646; repeated measures two-way ANOVA followed by Sidak’s comparison test, **P < 0.01; ***P < 0.001).

To assess which LDTg cell types are involved in the regulation of the freezing response, we individually silenced each neuronal population. We therefore injected a Cre-dependent AAV-hSyn-DIO-hM4-mCherry in vGlut2-Cre, ChAT-Cre or vGAT-Cre transgenic mouse lines, enabling the selective manipulation of glutamate, cholinergic or GABAergic neurons respectively. The fear response induced by electric shocks in CNO-treated LDTg^GluT-hM4^ and LDTg^ChAT-hM4^ mice was comparable to that of saline-treated controls (Fig. 2a, b, respectively). In striking contrast, freezing was markedly reduced in CNO-injected LDTg^GABA-hM4^ mice (Fig. 2c right panel), pinpointing to a key role of LDTg GABAergic transmission in electric shock-elicited freezing responses.

### Electric shocks sensitise GABAergic projections to the VTA that are required for freezing

**Fig. 3:**
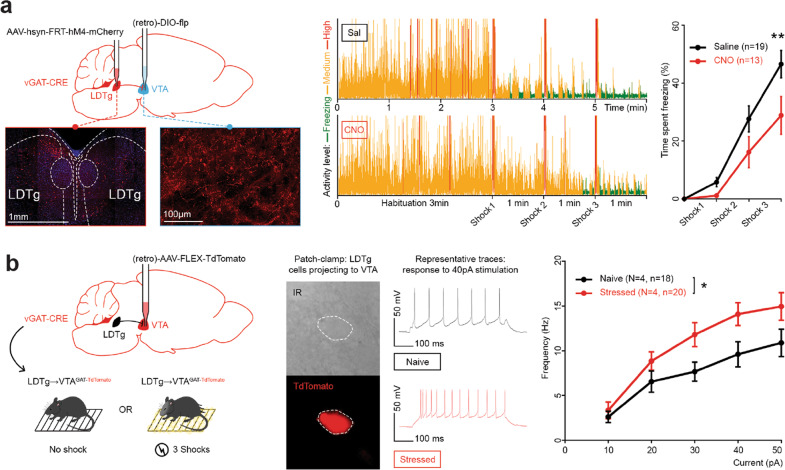
LDTg GABAergic projections to VTA control freezing and are sensitised by acute stress. **a** Silencing GABAergic LDTg projections to VTA reduces freezing. Left panel: vGAT-cre mice received injections of AAV-hsyn-FRT-hM4-mCherry in LDTg and retrograde DIO-flp in VTA then followed the same protocol as Fig. 1: microscopy images show mCherry-expressing cell bodies (injection site) and fibres in VTA. Middle panel: Representative traces showing activity of mice during the test. Right panel: Percentage of time mice spent freezing on each interval. Interaction treatment × shocks *F* (3, 90) = 3.088; repeated measures two-way ANOVA followed by Sidak’s comparison test, **P < 0.01). **b** GABAergic LDTg projections to VTA are sensitised by acute stress. Left panel: vGAT-cre mice were injected with retrograde AAV-FLEX-tdTomato in VTA then received either 3 shocks (Stressed) or no shock (Naive). Middle left panel: Microscopy images of GABAergic cell projecting to VTA in LDTg with transmission light (top) or emitting red fluorescence (bottom). Middle right panel: Representative voltage traces responses to a 40 pA current injection for each condition. Right panel: excitability profile of LDTg GABAergic neurons projecting to VTA in control or stressed mice. Plots depict action potential frequency after increasing current injection steps. Interaction treatment × current *F* (4, 144) = 1.866); Treatment factor *F* (1, 36) = 5.082; repeated measures two-way ANOVA followed by Sidak’s comparison test, *P < 0.05).

The previous results showed that independent silencing of either GABAergic LDTg neurons or LDTg^→VTA^ projections is sufficient to dampen the elicited freezing response. To test whether LDTg GABA neurons projecting to the VTA may be key in this process, we next manipulated the LDTg in a neurotransmitter- and projection-specific manner using a double intersectional strategy. We first injected in the VTA of vGAT-Cre mice a retrograde AAV-DIO-flp that allows the expression of the flippase (flp) in a Cre-dependent manner (i.e. in GABAergic LDTg^→VTA^ neurons). Next, a flippase-dependent inhibitory DREADD (AAV-hSyn-FRT-hM4-mCherry) was injected in the LDTg. While saline-treated LDTg^GABA→VTA-hM4^ mice exhibited a normal freezing response, CNO-treated mice demonstrated a significant downward shift of the freezing curve (Fig. 3a). This provides strong evidence of the key regulatory role of this GABAergic projection over the fear response.

Can exposure to aversive foot shocks alter the cellular properties of LDTg^→VTA^ GABAergic neurons in order to promote fear responses? We performed ex vivo whole-cell patch-clamp recordings in virally-tagged LDTg^→VTA^ GABAergic neurons. For this, vGAT-Cre mice were injected in the VTA with a retrograde AAV-FLEX-tdTomato (Fig. 3b). Mice were submitted to 3 consecutive electrical foot shocks as described for the behavioural testing, and recorded immediately after. Control mice were exposed to the chamber without receiving foot shocks. Exposure to foot shocks triggered an increased excitability of GABAergic LDTg^→VTA^ neurons evidenced by a higher discharge frequency to depolarising current injections when compared to non-shocked mice (Fig. 3b, right panel). Importantly this adaptation was not accompanied by any significant effect on passive membrane properties of the cell such as membrane resistance, membrane capacitance or resting membrane potential (Fig. S3a, b, c). To test whether electric shocks could alter the activity of other LDTg cell types we applied the same procedure to ChAT-Cre and vGluT2-Cre mice and recorded cholinergic and glutamatergic LDTg^→VTA^ neurons. In contrast to what we observed for projecting LDTg GABA neurons, electrical shocks failed to modify the excitability profile of cholinergic and glutamatergic neurons projecting to the VTA (Supplementary Fig. 5a). However, projecting GABAergic LDTg neurons do not only target the VTA. Hence, we last tested whether electrical shocks could alter the function of GABAergic neurons independently of the targeted brain regions. For this, vGATCre mice were injected in the vlPAG with a retrograde AAV-FLEX-tdTomato. Whereas we found an enhanced sensitivity of GABAergic LDTg^→VTA^ neurons, the excitability profile of GABAergic LDTg^→vlPAG^ neurons remained unaltered (Supplementary Fig. 5b). Thus, foot shocks affect the activity of a discrete GABAergic LDTg pathway.

### Optogenetic stimulation of LDTg GABA terminals within the VTA drives freezing

**Fig. 4:**
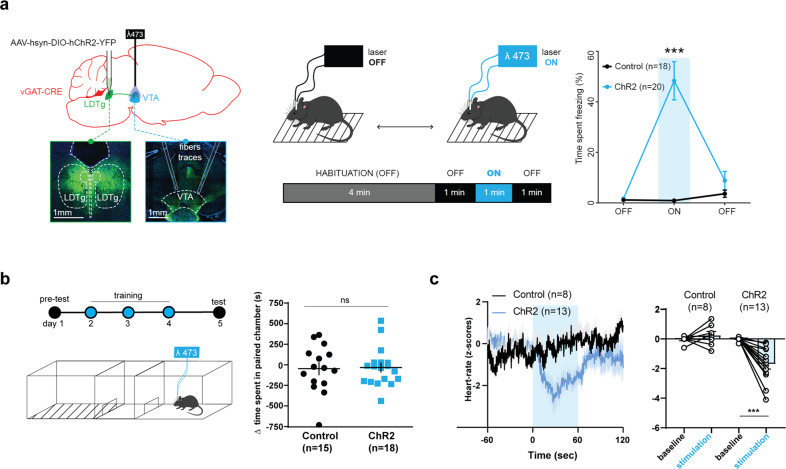
Activation of LDTg GABAergic inputs to the VTA induce freezing in absence of aversive experience. **a** Optogenetic in vivo activation of GABAergic LDTg terminals within the VTA induces spontaneous freezing. Left panel: vGAT-cre mice were injected with either AAV-hsyn-DIO-hChR2-YFP (ChR2) or AAV-hsyn-DIO-YFP (control) in LDTg and implanted with optic fibres above the VTA; microscopy images show green fluorescence at injection site and YFP expression at injection site and YFP-expressing fibres with optic fibres traces at implantation site in the VTA. Middle panel: Experimental timeline. Right panel: Percentage of time mice spent freezing depicted as mean ± S.E.M. on the following intervals (Interaction group × light *F* (2, 30) = 4.166; repeated measures two-way ANOVA followed by Sidak’s comparison test, ***P < 0.001). **b** Control and ChR2 mice received light stimulation in the paired compartment and no light stimulation in the unpaired side. Following 3 pairings on each side mice were allowed to freely explore the conditioning apparatus. Optogenetic in vivo activation of GABAergic LDTg terminals failed to induce place aversion (*t*_31_ = 0.1606, *P* = 0.8735). **c** Using telemetry monitoring of heart rate in anaesthetised mice, light stimulation in ChR2 mice elicited bradycardia that was not observed in control animals when laser was turned ON (*t*_7_ = 1.128, *P* = 0.2966 control; *t*_12_ = 5.191, *P* < 0.001 ChR2).

Our data gathered so far indicate that electric shocks trigger freezing by priming LDTg GABAergic LDTg^→VTA^ neurons. In order to test whether freezing could be induced without an aversive experience, we employed selective optogenetic stimulation in freely-behaving mice. We injected a Cre-dependent AAV-hsyn-DIO-hChR2-YFP in the LDTg of vGAT-Cre mice and bilaterally implanted optic fibres above the VTA. Light stimulation of ChR2 expressed on LDTg GABAergic terminals in the VTA was sufficient to induce significant freezing in absence of any aversive stimulus (Fig. 4a). Mice stopped freezing once the stimulation was turned off, indicative of a dynamic cellular process. Importantly, when mice were re-exposed to the same context 24 h after in the absence of light stimulation, no conditioned freezing was observed (Supplementary Fig. S6a). Additionally, we evaluated whether pairing light stimulation in a defined context could trigger conditioned place aversion. Three daily pairings were done and mice were tested in absence of light stimulation. Of note, the distance travelled by ChR2 mice in the unpaired chamber was higher than in the paired chamber as expected, where light stimulation induced immobility (Supplementary Fig. 6b). Nevertheless, both control and ChR2 mice spent similar amount of time in the paired chamber during the pre-test and test sessions (Fig. 4b). This indicates that activation of LDTg GABAergic terminals in the VTA does not produce aversive memories.

To differentiate whether optogenetic stimulation elicited unconditioned freezing or purely a motor arrest, we performed telemetric measurements of heart rate. This was done in anaesthetised mice to avoid confounding changes in heart function and physical activity. Light stimulation in ChR2 mice resulted in a significant heart-rate deceleration (i.e. bradycardia), which was not observed in control mice (Fig. 4c). Overall, this optogenetic experiment mimics two hallmarks of unconditioned freezing, namely immobility and bradycardia. Nevertheless, since this intervention does not trigger fear memories, and freezing being the sum of different physiological responses, including halted motor activity, we speculate that this artificial circuit recruitment may not fully recapitulate emotional responses to threatful stimuli.

### Optogenetic stimulation of LDTg GABA terminals in the VTA activates the amygdala

**Fig. 5:**
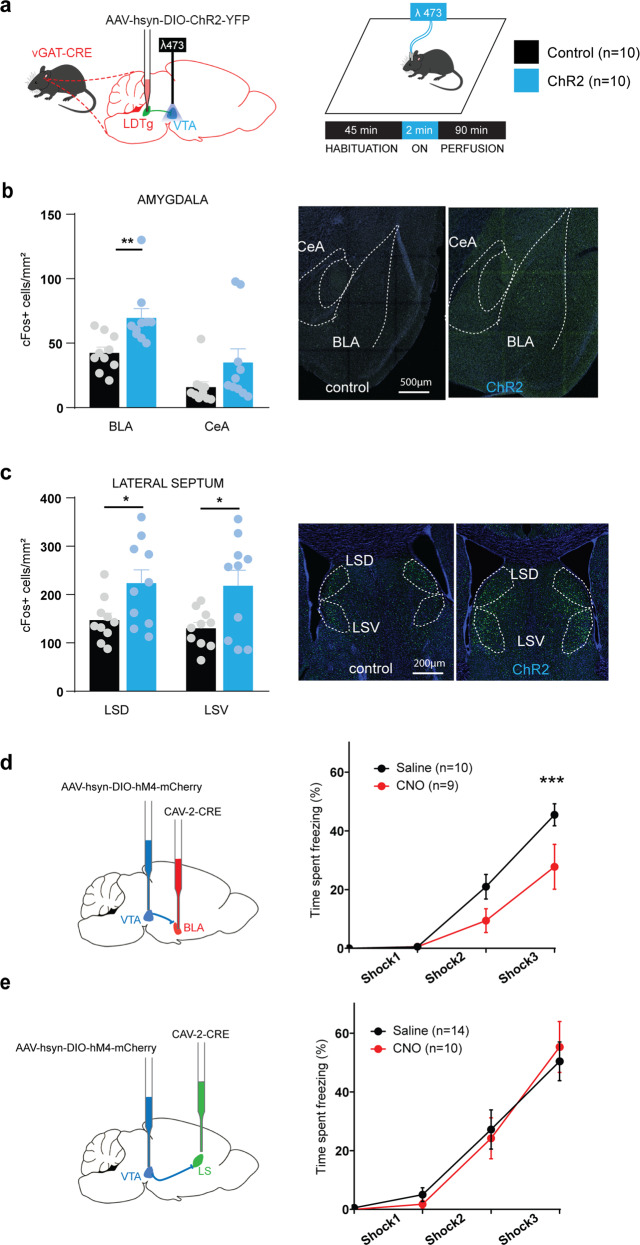
Stimulation of GABAergic LDTg terminals within the VTA triggers amygdala activation required for freezing. **a** Control and vGAT-Cre ChR2 mice were sacrificed 90 min after light stimulation as depicted in the experimental timeline (left panel). cFos-positive neurons were counted in (**b**) the basolateral and central amygdala (BLA and CeA respectively), (**c**) the dorsal and ventral part of the lateral septum (LSD and LSV respectively). **d** In WT mice, chemogenetic silencing of VTA projections to the BLA (Interaction treatment × shocks *F* (3, 51) = 3.604; repeated measures two-way ANOVA followed by Sidak’s comparison test, **P < 0.01), reduces freezing. **e** Chemogenetic silencing of VTA projections to the LS does not modify freezing behaviour (Interaction treatment × shocks *F* (3, 66) = 0.3606; repeated measures two-way ANOVA followed by Sidak’s comparison test, *P* = 0.7817). *P < 0.05; **P < 0.01.

Although some studies implicated the VTA in the formation of conditioned-freezing responses [44, 45], its role in unconditioned immediate freezing to threat as well as its output targets are unknown. To understand the circuits mediating LDTg GABA freezing response, we used optogenetic stimulation of LDTg GABA fibres over the VTA as before, and mice were sacrificed 90 min after light stimulation to conduct brain mapping of cFos expression (Fig. 5a). We have narrowed down our analyses to two brain structures known to play major roles in freezing, the amygdala [11] and the lateral septum [46]. As a major target of the VTA and considering its role in motor responses, we also analyzed the reactivity of the nucleus accumbens (NAc). Activation of LDTg GABA terminals within the VTA elicited a striking activation in both the amygdala and lateral septum but not in the NAc (Fig. 5b, c and Supplementary Fig. 7a, b respectively).

In light of these results, we next tested whether modulating VTA^→BLA^ or VTA^→LS^ projections would affect freezing responses. Using an intersectional viral strategy, we expressed an inhibitory DREADD in VTA^→BLA^ (Fig. 5d) or in VTA^→LS^ (Fig. 5e) neurons and exposed mice to electric foot shocks. A significant decreased in freezing was only observed when silencing VTA^→BLA^ projections but not VTA^→LS^ pathway (Fig. 5d, e respectively). These results point to the specificity for discrete VTA targets in the freezing response.

### Connectivity of GABA LDTg inputs to VTA cell types compared to VTA^→BLA^ neurons

Do LDTg GABAergic neurons directly modulate VTA^→BLA^ projecting neurons or do they encroach on VTA micro-circuits? To test this hypothesis, we probed for functional connectivity between this LDTg inhibitory input and the three VTA cell types (i.e. GABA, DA and Glu neurons) as well as VTA projecting neurons as described in Methods. Following photostimulation of LDTg terminals we found 24% of cells (7/29) connected with VTA^→BLA^ projecting neurons (Supplementary Fig. 8a). In striking contrast, 91% of VTA GABA cells (20/22) received GABA inputs from the LDTg (Supplementary Fig. 8b). These outward currents were blocked by picrotoxin, indicating that this response was through GABA-A receptors (Supplementary Fig. 8f). Also, GABAergic LDTg neurons made functional contacts with putative 95% of VTA DA neurons (19/20). Instead, only 33% of VTA Glu neurons (7/21) received GABA inputs from the LDTg (Supplementary Fig. 6d). Amongst the connected cells, the amplitude of optical inhibitory post synaptic currents (oIPSCs) was larger in VTA GABA and DA neurons when compared to VTA Glu cells or VTA^→BLA^ projecting neurons (Supplementary Fig. 6e). This first set of data indicates a more pronounced functional connectivity with the VTA micro-circuits over projecting ones, with a preference for DA and GABA VTA neurons.

### GABA LDTg inputs affect VTA inhibitory signalling controlling freezing

**Fig. 6:**
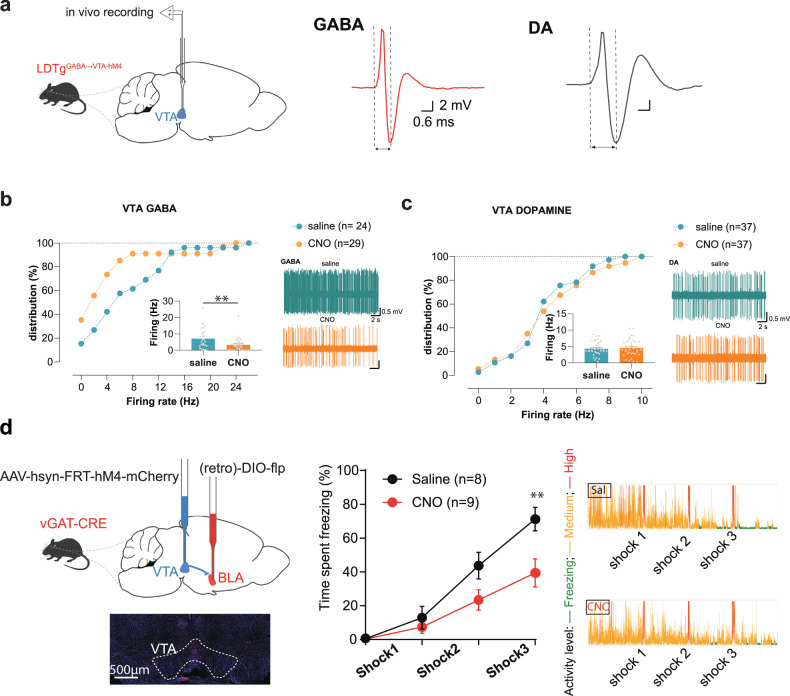
The LDTg is an upstream regulator of the VTA^→BLA^ pathway, which controls freezing. **a** In vivo recordings were performed in the VTA of anaesthetised LDTg^GABA→VTA-hM4^ mice and the activity of putative VTA GABA or DA neurons was analysed upon systemic administration of saline or CNO. The cumulative probability distribution of the firing rate, as well as the firing frequency (inset) are presented for putative VTA GABA (**b**) and VTA DA neurons (**c**). Representative traces for each experimental condition are presented. Silencing of LDTg GABAergic inputs to VTA selectively decreases the activity of VTA GABA neurons without altering VTA DA firing. **P < 0.01 Mann–Whitney test. **d** vGATCre mice received injection of AAV-hSyn-FRT-hM4-mCherry in the VTA and retro AAV-DIO-Flp in the BLA, and then underwent freezing paradigm. Microscopy image shows mCherry expression at VTA injection site. Selective silencing of GABAergic VTA^→BLA^ neurons diminished freezing responses (Interaction treatment × shocks *F* (3, 45) = 4.947; repeated measures two-way ANOVA followed by Sidak’s comparison test, **P < 0.01.

Results from the connectivity analysis suggest that silencing GABAergic LDTg^→VTA^ neurons should preferentially modulate VTA GABAergic or DA neurons activity. To test this possibility, and to have a more comprehensive view of LDTg control over VTA function, we performed in vivo recordings in anesthetised animals while silencing GABAergic LDTg^→VTA^ projections (Fig. 6a). In saline-treated mice, putative GABAergic VTA neurons exhibited a classical firing pattern of activity (Fig. 6b) similar to previous reports [34–36]. In contrast, CNO-treated mice exhibited a strong leftward shift of the firing rate distribution of putative VTA GABA neurons, reflecting a marked decreased in activity (Fig. 6b). To determine whether silencing GABAergic LDTg^→VTA^ pathway had a broader impact onto VTA homoeostasis, we additionally recorded in vivo the activity of VTA DA neurons. The firing rate distribution (Fig. 6c), the bursting activity and the four main modes of firing patterns of VTA DA neurons [47] did not differ between saline- and CNO-treated groups (Supplementary Fig. 9).

VTA GABA neurons can be long-range neurons or they could modulate VTA function via local connectivity [34, 35, 48–50]. To try to solve this issue we sought to identify the nature of the neurotransmitter conveying freezing-related information to the amygdala. We therefore silenced in a projection- and neurotransmitter-specific manner DA, Glu and GABA VTA^→BLA^ neurons while submitting mice to electric foot shocks to elicit freezing. Only the silencing of GABAergic VTA^→BLA^, but not DA or Glu, neurons decreased freezing (Fig. 6d, Supplementary Fig. 10a, b, respectively).

Overall, this set of data link an inhibitory input from the LDTg to VTA GABA function and provides evidence of a coordinated response of LDTg-VTA-amygdala macro-circuit during aversive processing (Supplementary Fig. 11).

## Discussion

While many brain regions have been identified to support freezing responses, the role that modulatory sites provide to this hardwired scaffold is not known. Our results provide a novel framework to understand how aversive experiences trigger rapid cellular changes ultimately leading to defensive behaviours. We show here that activity of LDTg GABAergic inputs to the VTA is necessary for the manifestation of freezing behaviours in response to imminent danger. Optogenetic stimulation of this projection is sufficient to promote freezing in absence of threat and recruitment of amygdala substrates. Our study demonstrates an uncovered role of this pathway in adaptive defensive behaviours to threat, which provides novel insights of stress-coping circuits.

### Macro- and micro-circuits of brain defensive network

The brain defensive network includes several interconnected cortical and subcortical areas essential for threat detection and processing in order to timely drive defensive responses [37, 51, 52]. Functional imaging studies in humans and extended molecular, cellular and circuit dissections in rodents positioned the amygdala, periaqueductal grey and prefrontal cortex as core actuators of the control of fear responses, which include freezing. While a large body of evidence provides solid ground for macro- and local-circuit governing conditioned-freezing responses [11], a detailed comprehension of the cellular mechanisms and sequence of events eliciting unconditioned freezing behaviour is still lacking. This is highly relevant since the symptoms and pathogenesis of threat-related disorders rely on both learned and innate fear responses [10, 53]. In our study we demonstrate that electric foot shocks trigger an immediate increase in excitability of inhibitory LDTg projecting neurons to the VTA. The silencing of this specific projection has selective impact onto the function of VTA GABAergic neurons, without altering the firing of VTA DA cells. Furthermore, we dissected VTA outputs and observed that silencing of VTA GABAergic projections to the amygdala dampens unconditioned freezing responses to foot shocks. In a recent study, selective optogenetic inhibition of VTA DA terminals in the amygdala impaired the acquisition of aversive memories but did not impact unconditioned freezing to foot shocks [54]. This suggest that different VTA to amygdala neurotransmitter populations contribute to unconditioned and conditioned freezing.

Supporting a role of the VTA in defensive responses, two recent reports linked VTA GABA and glutamatergic neurons with innate defensive behaviour [48, 55]. Indeed, exposure to predator odour or to looming stimulus elicited a transient activation of VTA glutamate neurons associated with escape behaviours [55]. Also, the onset of looming-evoked escape behaviours correlated with Ca^2+^ transients in VTA GABA neurons [48]. In accordance with our study, the authors reported that direct optogenetic stimulation of this global neuronal population produced freezing followed by flight-to-nest behaviour. This clearly reveals an emerging role for the VTA in escape defensive behaviours. Yet, although VTA neurons have been shown to react to foot shocks [34], their involvement in eliciting rapid fear responses remained uncovered. Here, we broadened the role of VTA in defensive responses by showing the triggering of freezing by solely modulating a GABA input from the pontine region. Studies addressing the role of the VTA in positive and negative valence have by far focussed onto the role of VTA DA neurons. Here, in the experiments we carried out, we could not implicate this neuronal population, but however, we identified a role for long-range VTA GABAergic neurons. This suggests that similarly to VTA DA neurons, GABAergic long-range neurons are likely to appear as, at least in part, segregated neuronal population computing distinct environmental stimuli and internal demands, in order to drive different facets of behavioural responses ranging from modulation of morphine rewarding properties, to associative learning and unconditioned freezing (present study) [49, 56, 57].

Overall, in these macro- and micro-circuits of brain defensive behaviours we revealed, one putative model would be that electric shocks activate GABAergic LDTg neurons to produce disinhibition of VTA^→BLA^ neurons via the modulation of local VTA neurons, putatively GABA [50].

### The LDTg-VTA axis: positive and negative valence

To navigate in complex and rapidly evolving environments, mammals must adapt their behaviours to gain access to vital resources while avoiding harmful situations. These behavioural responses are finely tuned by both rapid and enduring cellular adaptations when facing rewarding and/or aversive challenges [58]. Dysregulation of this homoeostatic balance leads to inappropriate choices and increases the risk of developing psychiatric conditions [59]. Historically, the LDTg-VTA axis has been heavily linked with reward processing and reinforcement [60, 61]. The LDTg is an important modulator of VTA dopamine firing activity and consequently forebrain dopamine release [20, 62, 63]. Pharmacological and lesions studies have highlighted its key role in attributing reward value to stimuli, and mediating several cellular and behavioural adaptations to addictive substances such as cocaine and nicotine [64]. Projection-specific manipulations revealed its contribution to appetitively motivated and rewarding behaviours [16, 43], with distinct contribution of cholinergic and glutamatergic LDTg projections to these processes [42]. Indeed, selective optogenetic stimulation in the VTA of LDTg glutamatergic or cholinergic terminals is rewarding, reflected by the number of time spent and entries into a stimulus-paired chamber [42]. Recent evidence point to a partial involvement of GABAergic inputs to the VTA in the reinstatement of cocaine seeking behaviours [65]. Despite these solid proofs of the LDTg-VTA axis involvement in positive stimuli processing, our present work calls into question this accepted view and demonstrates a causal implication of LDTg inhibitory inputs to the VTA in processing immediate aversive challenges. Our results are supported by evidence showing that optogenetic manipulation of local LDTg interneurons impact innate fear induced by olfactory cues [21]. This is likely to affect LDTg outputs and modulate downstream target activity, including the LDTg-VTA axis identified here. Also, our previous work demonstrated that overactivation of LDTg cholinergic inputs to VTA dopamine neurons following social aggression triggered the appearance of depressive-like symptoms [20]. More recently, a neuron-derived trophic molecule, neuregulin-1, has been shown to be increased in the LDTg following chronic social defeat stress and to promote depressive-like behaviours by impinging on VTA DA neurons activity [66]. Last, in utero exposure to the stress hormones glucocorticoids induces motivational deficits, which can be counteracted by modulating LDTg-VTA projections [67]. Altogether, this suggests that salient environmental stimuli, either rewarding or aversive, would recruit independent populations of LDTg neurons, and that the early polarisation of positive and negative networks was an oversimplification [68]. Thus, cholinergic/glutamatergic and GABAergic LDTg neurons might encode complementary motivational states, promoting approach or defence, respectively, via projections to the VTA. Approach and defence responses require the coordinated involvement of motor centres. In particular, the pedunculopontine nucleus (PPN), a closely related centre in the brainstem, sends ascending projections to most basal ganglia nuclei, notably the substantia nigra (SN), controlling motor responses [23]. For example, optogenetic activation of PPN cholinergic terminals within the SN increases locomotion [17]. A recent bioRxiv report shows that GABAergic PPN neurons make synapses onto SN DA neurons, and that their activation impairs exploratory locomotion and halts movement initiation, but did not produce freezing responses [69]. Overall, distinct GABAergic projections from the LDTg and PPN to the VTA and SN, respectively, are likely to convey complementary signals driving fear and motor responses. Future work is needed to understand a potential role of the PPN^→SN^ GABAergic pathway in response to aversive stimuli such as foot shocks, and a putative crosstalk between these parallel brainstem projections.

## Conclusions

Uncovering modulatory regions like the one described here is paramount to understand natural responses to threat stimuli. Importantly, the pathway identified here was restricted to the induction of unconditioned freezing and did not produce aversive memory formation. Furthermore, understanding synaptic and cellular adaptations on these circuits might help us understand the underlying mechanisms contributing to symptoms of pathological conditions. Future studies will need to identify the molecular actuators driving these circuits (mal)adaptations in order to target them with classic pharmacological drugs. Alternatively, future therapeutic approaches involving brain modulation techniques might be able to capitalise from the present findings.

## Supplementary information

Supplementary Figure Legends

Fig.S1

Fig.S2

Fig.S3

Fig.S4

Fig.S5

Fig.S6

Fig.S7

Fig.S8

Fig.S9

Fig.S10

Fig.S11

Activation of LDTg GABAergic terminals within the VTA elicits freezing
