# Supplementary material for: A non-canonical GABAergic pathway to the VTA promotes unconditioned freezing
Source: Mol Psychiatry. 2022 Sep 20;27(12):4905–17. doi: 10.1038/s41380-022-01765-7 (PMC9763111; doi:10.1038/s41380-022-01765-7)
Supplement: Supplementary file 1 — Supplementary Figure Legends [file 41380_2022_1765_MOESM1_ESM.docx]

**SUPPLEMENTARY FIGURES LEGENDS**

**A non-canonical GABAergic pathway to the VTA promotes unconditioned freezing**

Loïc Broussot ^1, 2*^, Thomas Contesse ^1, 2*^, Renan Costa-Campos^1, 2^, Christelle Glangetas^3^, Léa Royon ^1, 2^, Hugo Fofo ^1, 2^, Thomas Lorivel ^2^, François Georges ^3^, Sebastian P. Fernandez ^1, 2, 4^ and Jacques Barik ^1, 2, 4^.

1 Université Côte d’Azur, Nice, France.

2 Institut de Pharmacologie Moléculaire & Cellulaire, CNRS UMR7275, Valbonne, France.

3 Université de Bordeaux, CNRS, IMN, UMR 5293, F-33000 Bordeaux, France.

4 Co-last and co-corresponding authors.

**Supplementary Figure 1. Silencing of LDTg does not alter aversive memory formation.** (**a**) Wild-type mice were injected with an AAV-hsyn-hM4-mCherry in the LDTg. Mice received either saline or CNO 30 min before receiving 3 consecutive electrical foot shocks. Mice were re-exposed in a drug-free state 24h to the same context without shocks. (**b**) Freezing responses did not differ between the 2 groups when re-exposed to the same context 24h after. DAY 1: Interaction treatment x shocks F(3,51) = 0.1732; Treatment factor F(1, 17) = 6.423; repeated measures two-way ANOVA followed by Sidak’s comparison test, * P<0.05. DAY 2: P=0.2839, t-test.

**Supplementary Figure 2. Selective silencing of LDTg projections to the CeA or vlPAG does not alter freezing.** Wild-type mice were injected with an AAV-hsyn-DIO-hM4-mCherry in the LDTg and a retrograde CAV-2-Cre in either (a) the central amygdala (CeA) or (b) ventrolateral periaqueductal gray (vlPAG). (**a**) Microscopy images show red fluorescence in LDTg and red fibres within the CeA. Silencing LDTg projections to CeA does not alter freezing. Interaction treatment x shocks F(3, 87) = 0.997; repeated measures two-way ANOVA followed by Sidak’s comparison test, P=0.3982. (**b**) Microscopy images show mCherry expression in LDTg and mCherry-expressing fibres within the vlPAG. Silencing LDTg projections to vlPAG does not alter freezing. Interaction treatment x shocks F(3, 63) = 1.592; repeated measures two-way ANOVA followed by Sidak’s comparison test, P=0.2001.

**Supplementary Figure 3. Examples of activity levels when silencing (or not) glutamatergic (2a), cholinergic (2b) or GABAergic (2c) LDTg neurons.**

**Supplementary Figure 4. Passive membrane properties of LDTg GABAergic neurons projecting to the VTA are unaffected by acute stress.** (a, b, c) Measure of input resistance, membrane capacitance and resisting membrane potential respectively in mice that received either three electrical foot shocks (stressed) or no shock (naïve) just before ex-vivo patch-clamp recording. All plots depict mean ± S.E.M. (**a**) Input resistance (P=0.2367, t-test). (**b**) Membrane capacitance (P=0.0694, Mann-Whitney test). (**c**) Resting membrane potential (P=0.0604, t-test).

**Supplementary Figure 5. Discrete cellular effects of foot-shocks.**

(**a**) Left panel: ChaT-Cre and vGluT2-Cre mice were injected with retrograde AAV-FLEX-tdTomato in the VTA then received either 3 shocks (Stressed) or no shock (Naive). Neither cholinergic (Middle panel) nor glutamatergic (Right panel) projections to VTA are affected by stress as shown by the similar profiles of excitability between the 2 conditions. (**b**) Left panel: vGAT-Cre mice were injected with retrograde AAV-FLEX-tdTomato in the VTA and treated as in ‘a’. Right panel: the excitability profile of LDTg GABAergic neurons projecting to the vlPAG do not differ between control or stressed mice. Statistical comparisons were made using repeated measures two-way ANOVA followed by Sidak’s comparison test, revealing no significant differences.

**Supplementary Figure 6. Effects of photoactivation of LDTg GABAergic terminals does on aversive memory and distance travelled.** (**a**) Control and ChR2 mice photo-stimulated in Fig. 2a were re-exposed to the same context 24h after. Data indicate an absence of conditioned freezing. (**b**) The mean of the distance travelled over the 3 days of conditioning has been measured in the paired and unpaired chambers for control and ChR2 mice. Repeated measures two-way ANOVA followed by Sidak’s comparison test, P=0.0672 for control and P<0.001 for ChR groups.

**Supplementary Figure 7. Stimulation of GABAergic LDTg terminals within the VTA does not induce activation of the NAc.** (**a**) Control and vGAT-Cre ChR2 mice were sacrificed 90 min after light stimulation as depicted in the experimental timeline. cFos-positive neurons were counted in the NAc core and shell subdivisions. No difference was found between the 2 conditions. (**b**) Example of freezing traces obtained when silencing or not VTA^🡪BLA^ projections (see Fig. 5d). (**c**) Example of freezing traces obtained when silencing or not VTA^🡪LS^ projections (see Fig. 5e).

**Supplementary Figure 8. Functional connectivity between LDTg GABAergic inputs and VTA cell substrates.** VTA slices were obtained from vGAT-Cre ChR2 mice injected either with a retro-AAV-tdTomato in the BLA (**a**) or AAV-hSyn-DIO-mCherry in the VTA (**b**) or no viral injection in the VTA (**c**). We also prepared slices from vGluT2-Cre mice injected with AAV-hSyn-DIO-mCherry in the VTA and AAV-hSyn-ChR2-YFP in the LDTg. *Ex vivo* recordings were obtained from tdTomato- or mCherry-expressing VTA neurons following light stimulation. Putative VTA DA neurons were identified as described in methods. The percentage of cells exhibiting optically-induced inhibitory postsynaptic currents (oIPSC) are represented in pie charts. (**e**) The mean current responses of VTA^🡪BLA^, VTA^GABA^, VTA^DA^ or VTA^Glu^ responding neurons is reported in pA. (**f**) Optically-induced inhibitory postsynaptic currents (oIPSC) were virtually abolished in the presence of picrotoxin (PTX, 50 μM). Right panel: Amplitude of oIPSCs in VTA^GABA^ or VTA^🡪BLA^ neurons.

**Supplementary Figure 9. Impact of silencing LDTg inhibitory inputs onto VTA DA neurons activity.** (**a**) vGAT-Cre mice were injected with an AAV-hSyn-DIO-hM4-mCherry in the LDTg and *in vivo* recordings were performed in the VTA in anesthetized mice. The activity of putative VTA DA neurons was analysed upon systemic administration of saline or CNO. (**b**) The bursting rate (left panel) and % of spikes in bursts (SIB, right panel) did not differ between treatments. (**c**) Analysis of VTA DA neurons activity based on both the firing and bursting patterns. LFLB: low firing, low bursting; HFLB: high firing, low bursting; LFHB: low firing, high bursting; HFHB: high firing, high bursting. The inhibition of LDTg GABAergic inputs to VTA does not alter the activity of VTA DA neurons.

**Supplementary Figure 10. Lack of effect of silencing Glu or DA VTA^🡪BLA^ neurons on freezing responses.** vGluT2-Cre (**a**) or DAT-Cre (**b**) mice were injected with a retrograde AAV-DIO-flp in the BLA and a flippase(flp)-dependent inhibitory DREADD in the VTA. Following exposure to electric shocks, freezing responses did not differ between saline- or CNO-treated mice. Interaction treatment x shocks F(3, 57) = 0.751, P=0.5261 for vGluT2 and F(3, 63) = 0.796, P=0.500 for DAT-Cre; repeated measures two-way ANOVA followed by Sidak’s comparison test.

**Supplementary Figure 11. Schematic model of the findings.**
