# Supplementary figures and images for: A non-canonical GABAergic pathway to the VTA promotes unconditioned freezing
Source: Mol Psychiatry. 2022 Sep 20;27(12):4905–17. doi: 10.1038/s41380-022-01765-7 (PMC9763111; doi:10.1038/s41380-022-01765-7)

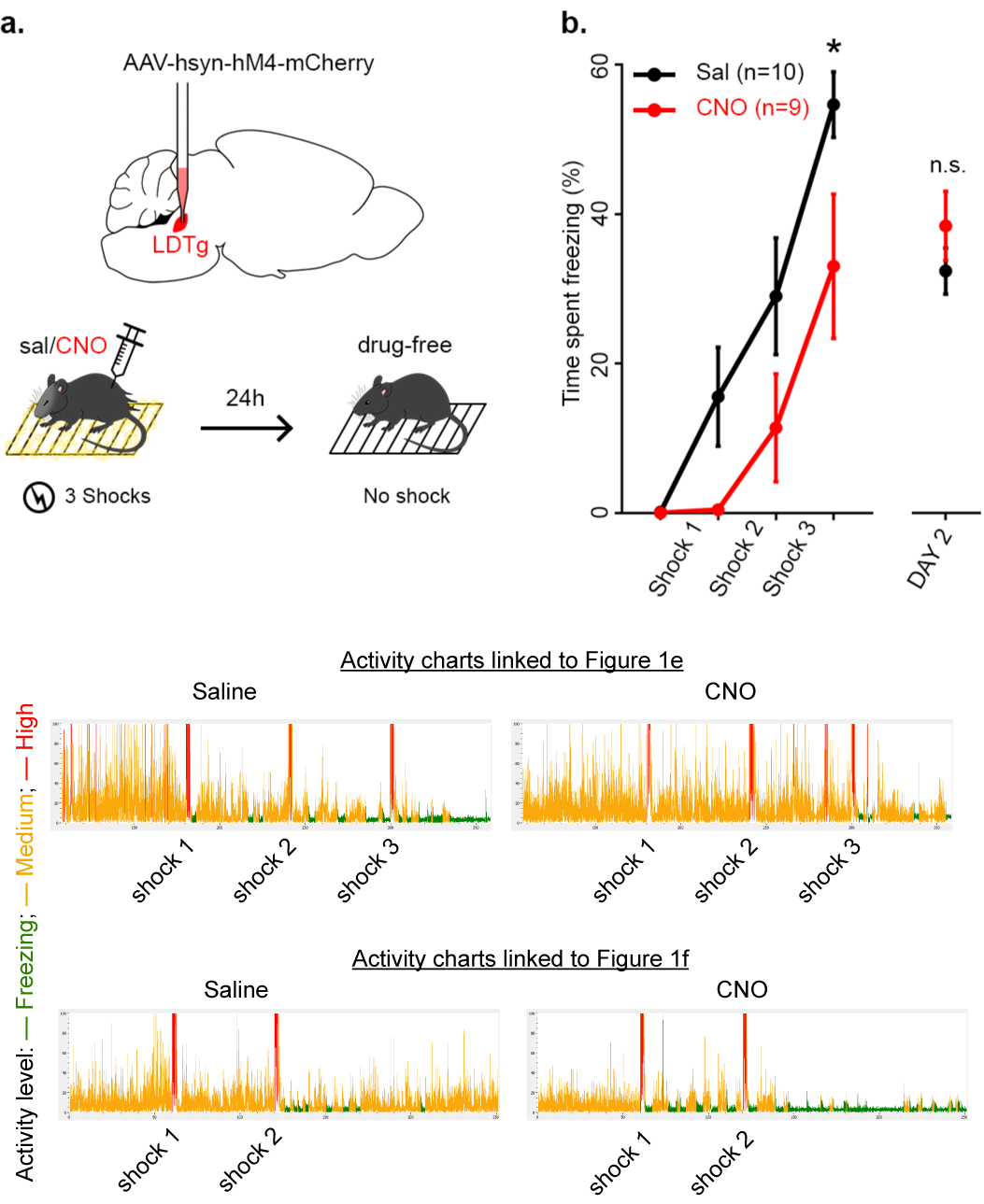

Supplement: Supplementary file 2 — Fig.S1 [file 41380_2022_1765_MOESM2_ESM.tif]

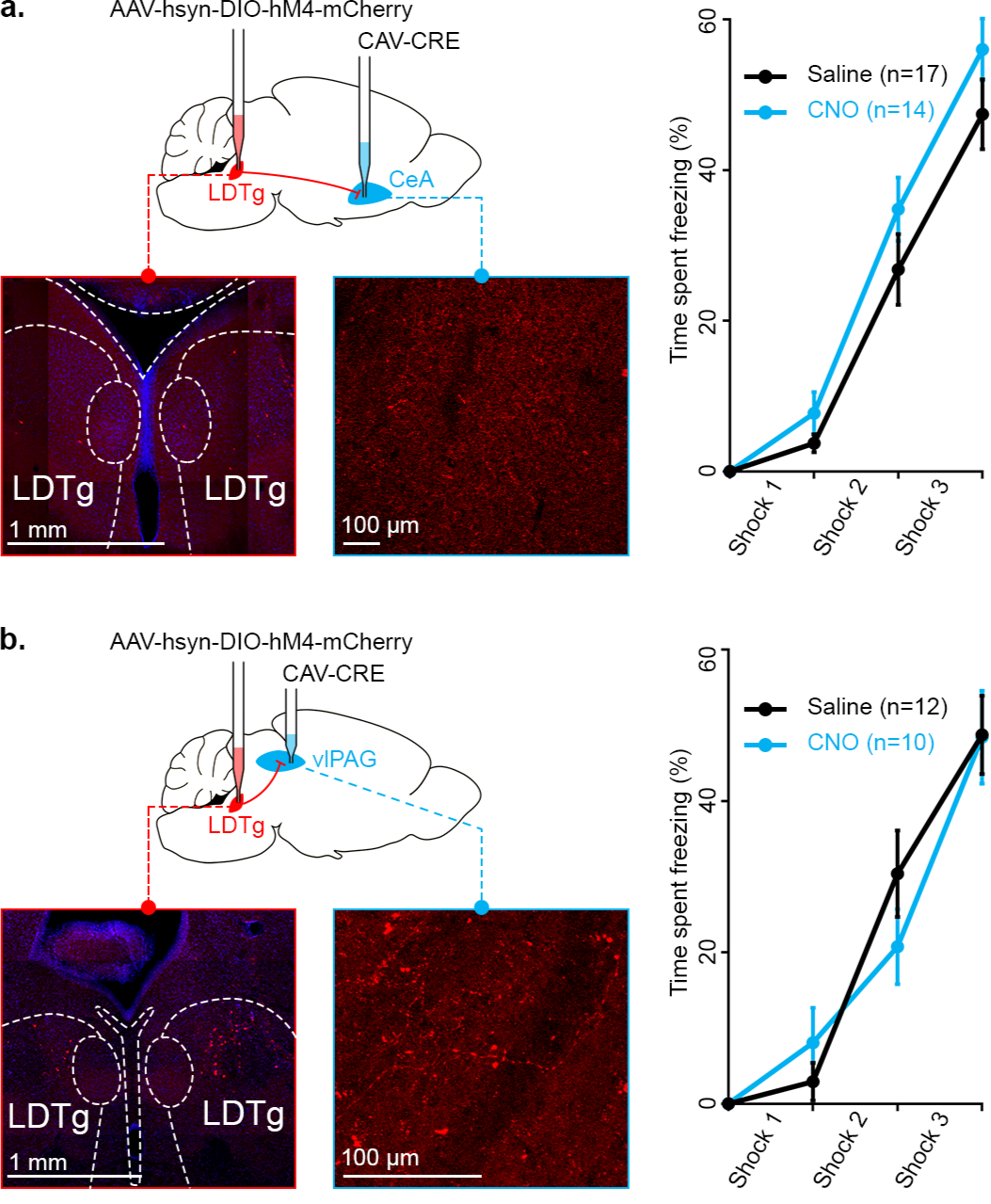

Supplement: Supplementary file 3 — Fig.S2 [file 41380_2022_1765_MOESM3_ESM.tif]

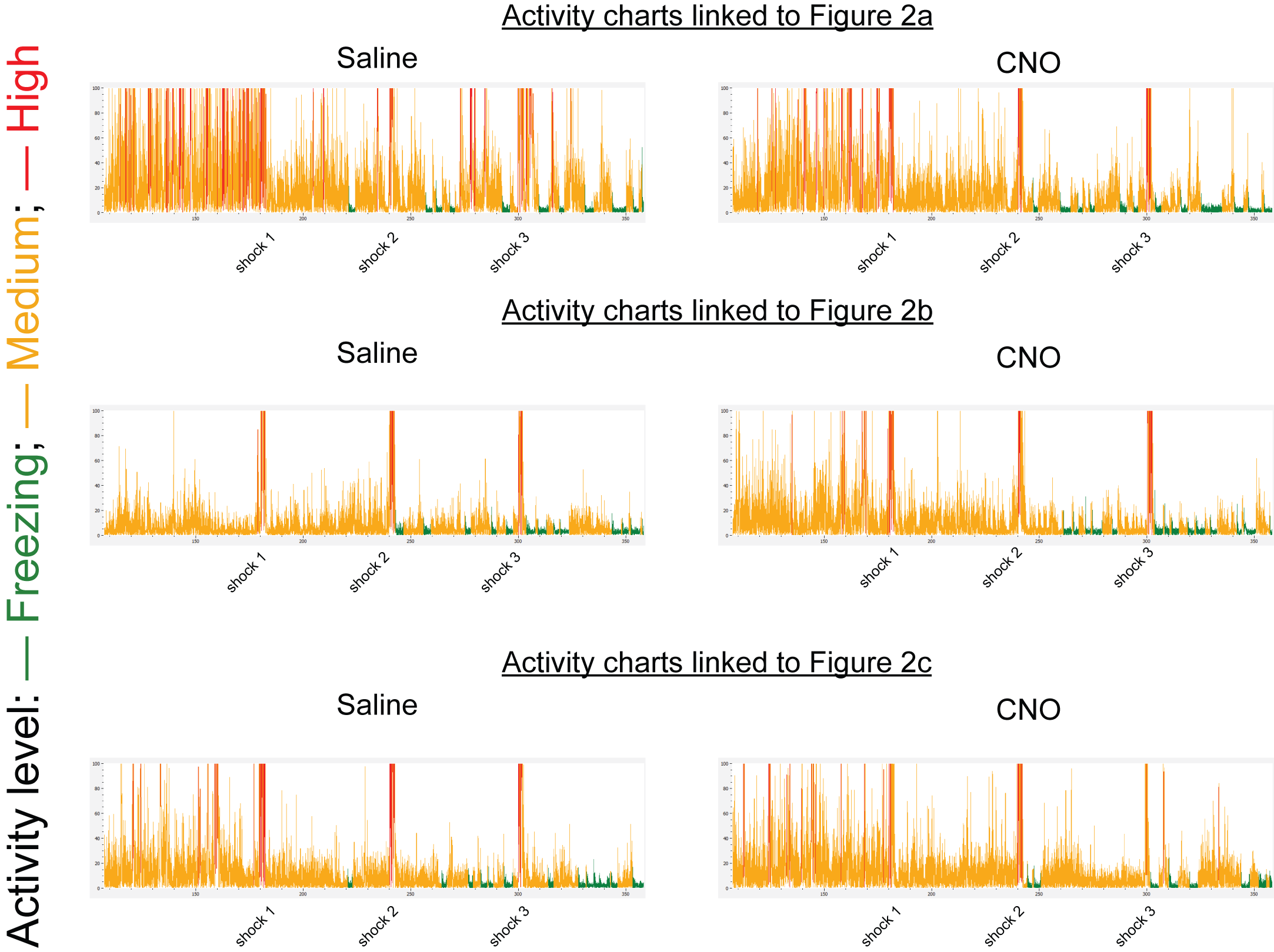

Supplement: Supplementary file 4 — Fig.S3 [file 41380_2022_1765_MOESM4_ESM.tif]

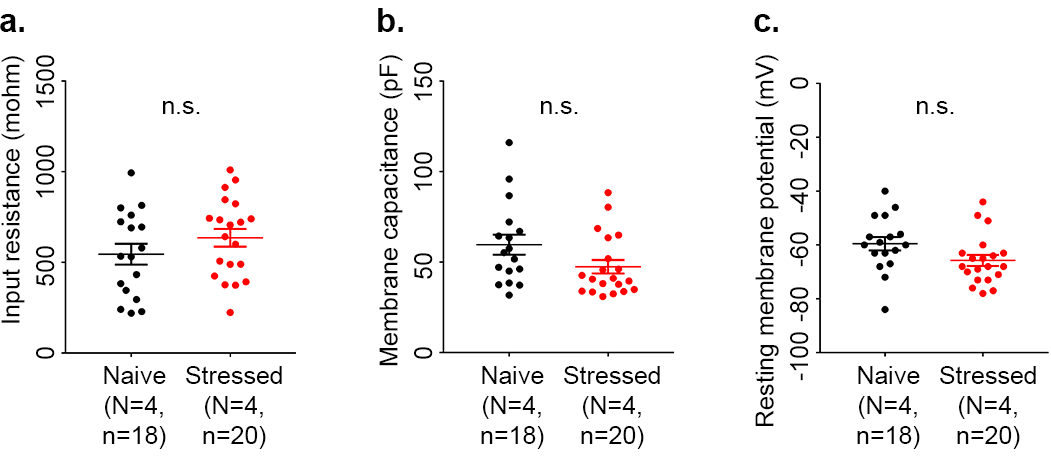

Supplement: Supplementary file 5 — Fig.S4 [file 41380_2022_1765_MOESM5_ESM.tif]

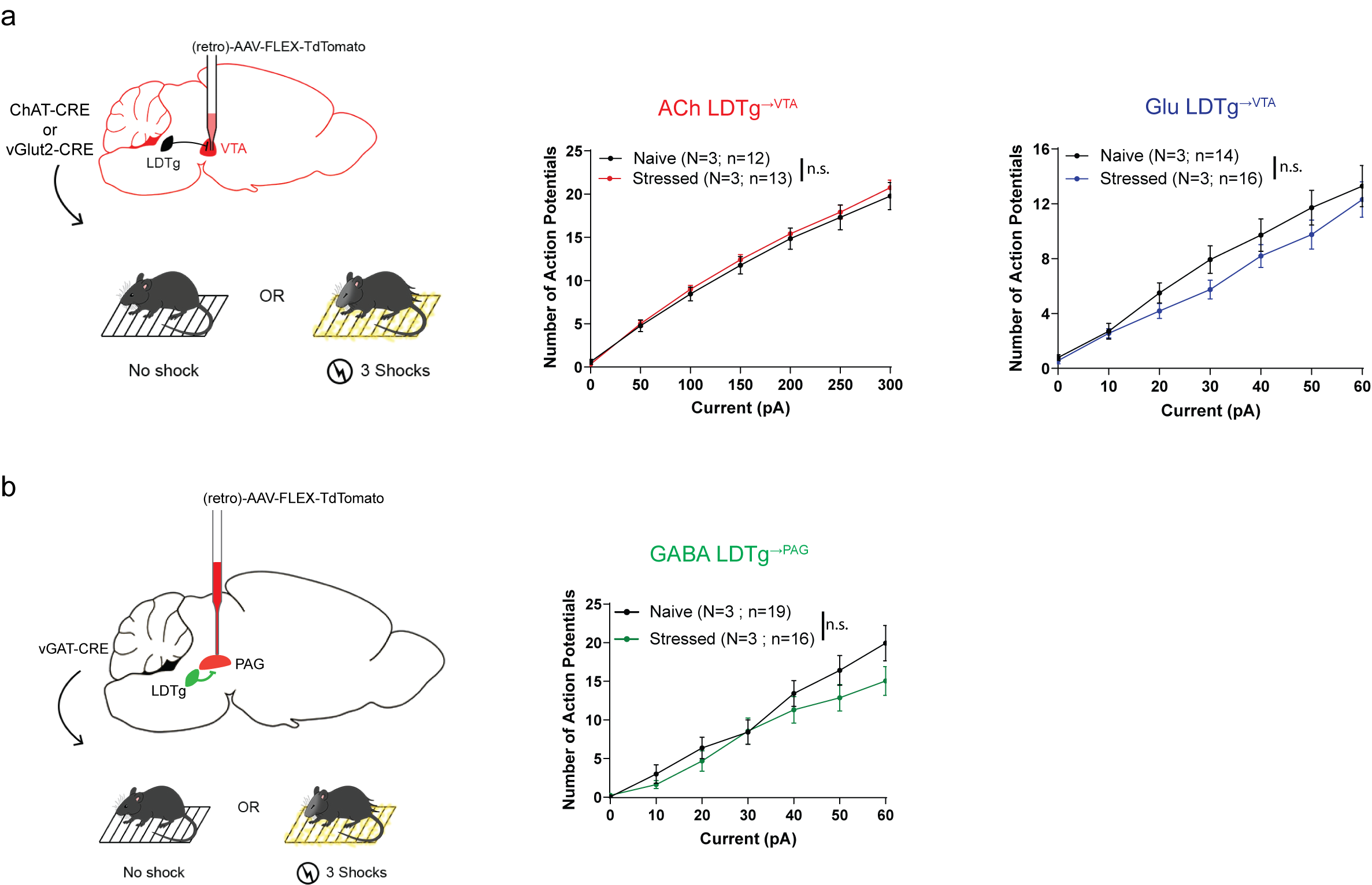

Supplement: Supplementary file 6 — Fig.S5 [file 41380_2022_1765_MOESM6_ESM.tif]

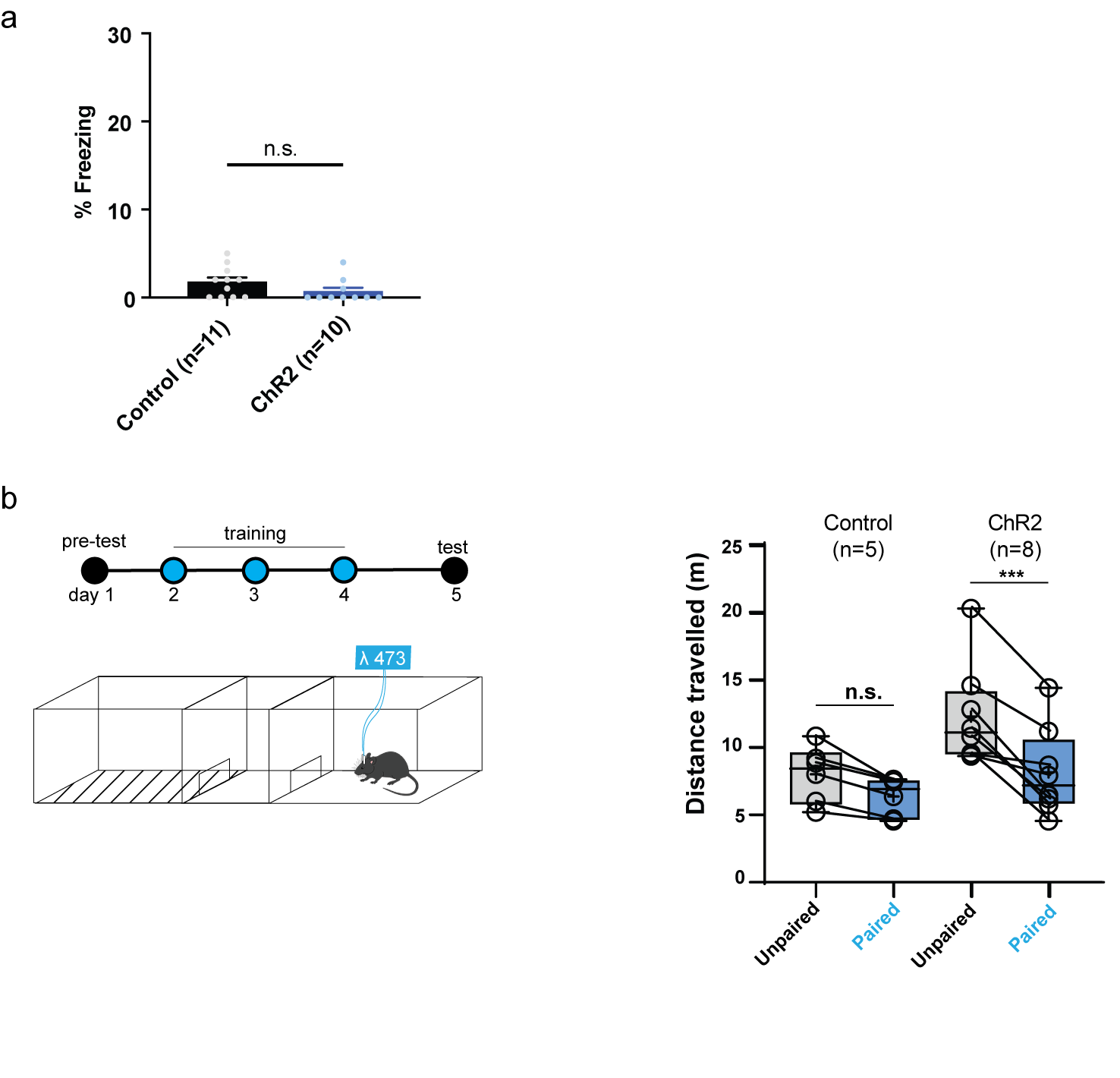

Supplement: Supplementary file 7 — Fig.S6 [file 41380_2022_1765_MOESM7_ESM.tif]

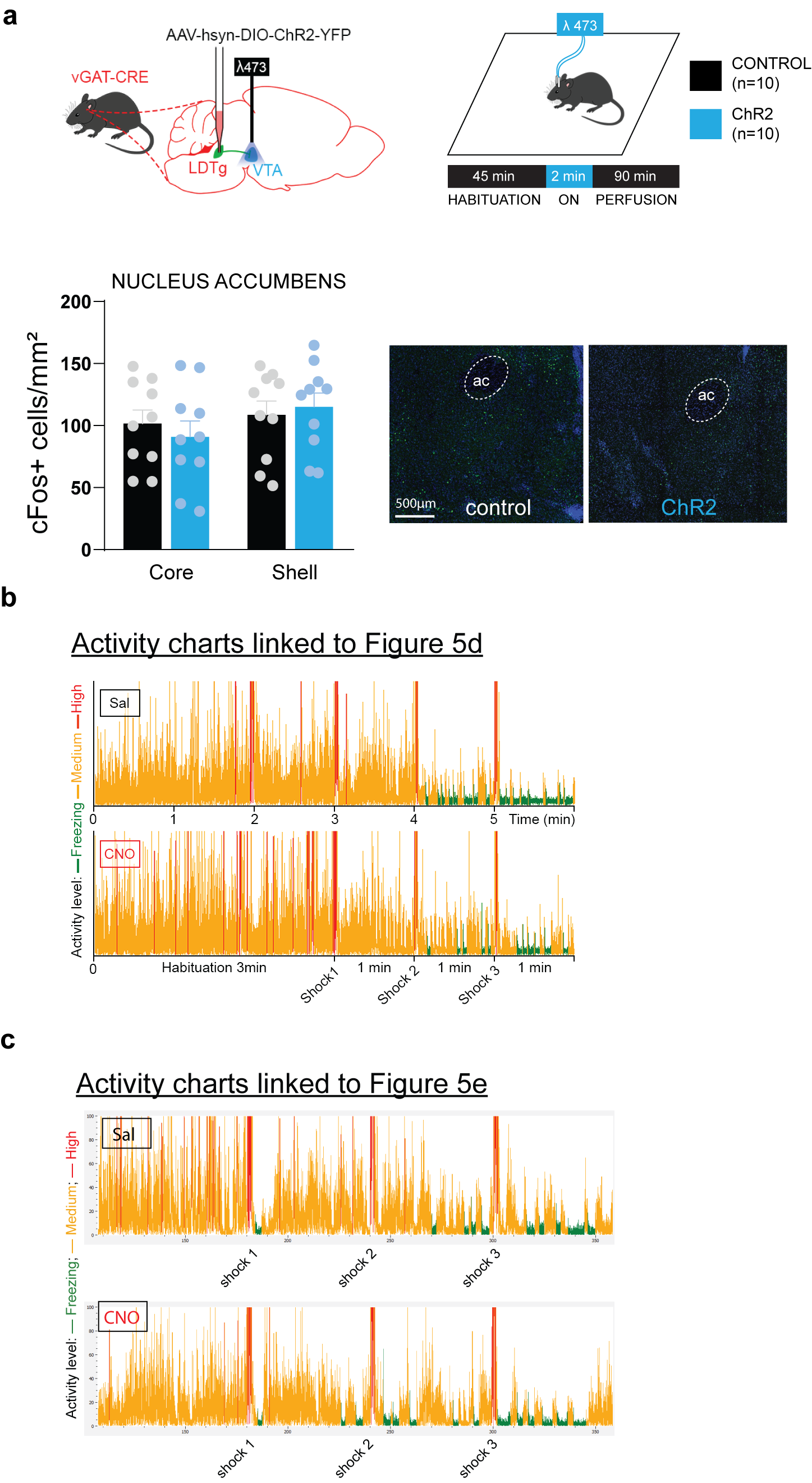

Supplement: Supplementary file 8 — Fig.S7 [file 41380_2022_1765_MOESM8_ESM.tif]

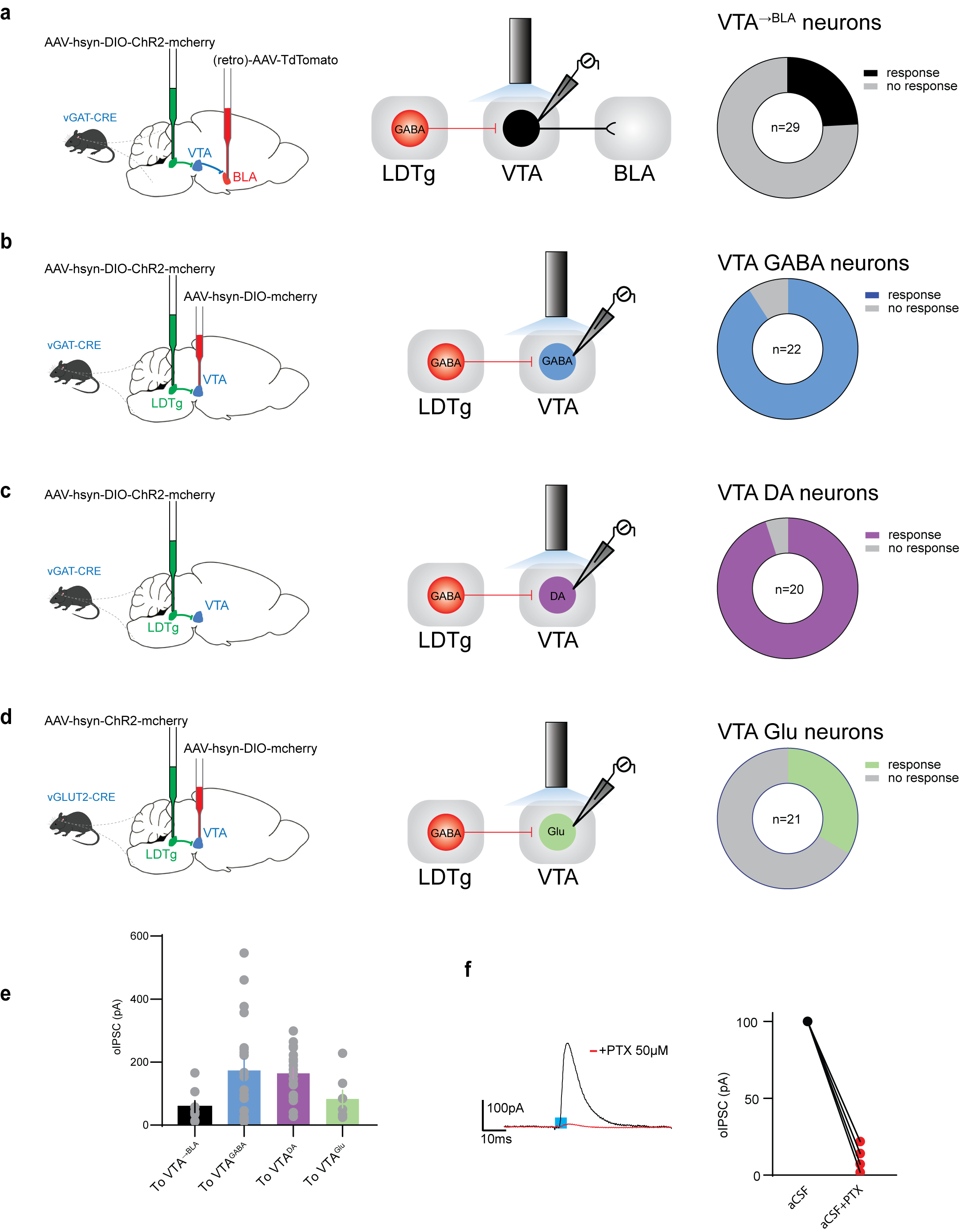

Supplement: Supplementary file 9 — Fig.S8 [file 41380_2022_1765_MOESM9_ESM.tif]

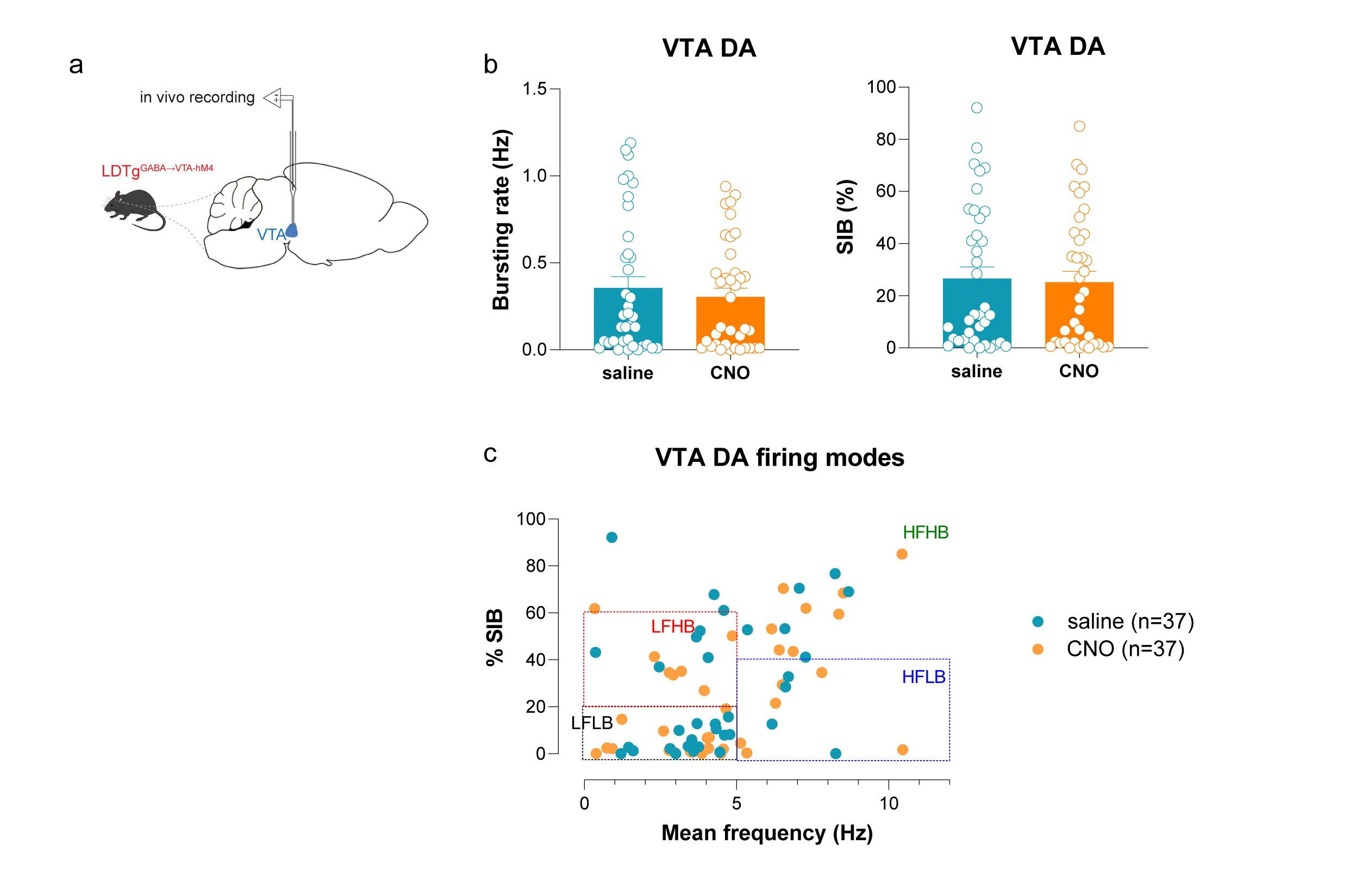

Supplement: Supplementary file 10 — Fig.S9 [file 41380_2022_1765_MOESM10_ESM.tif]

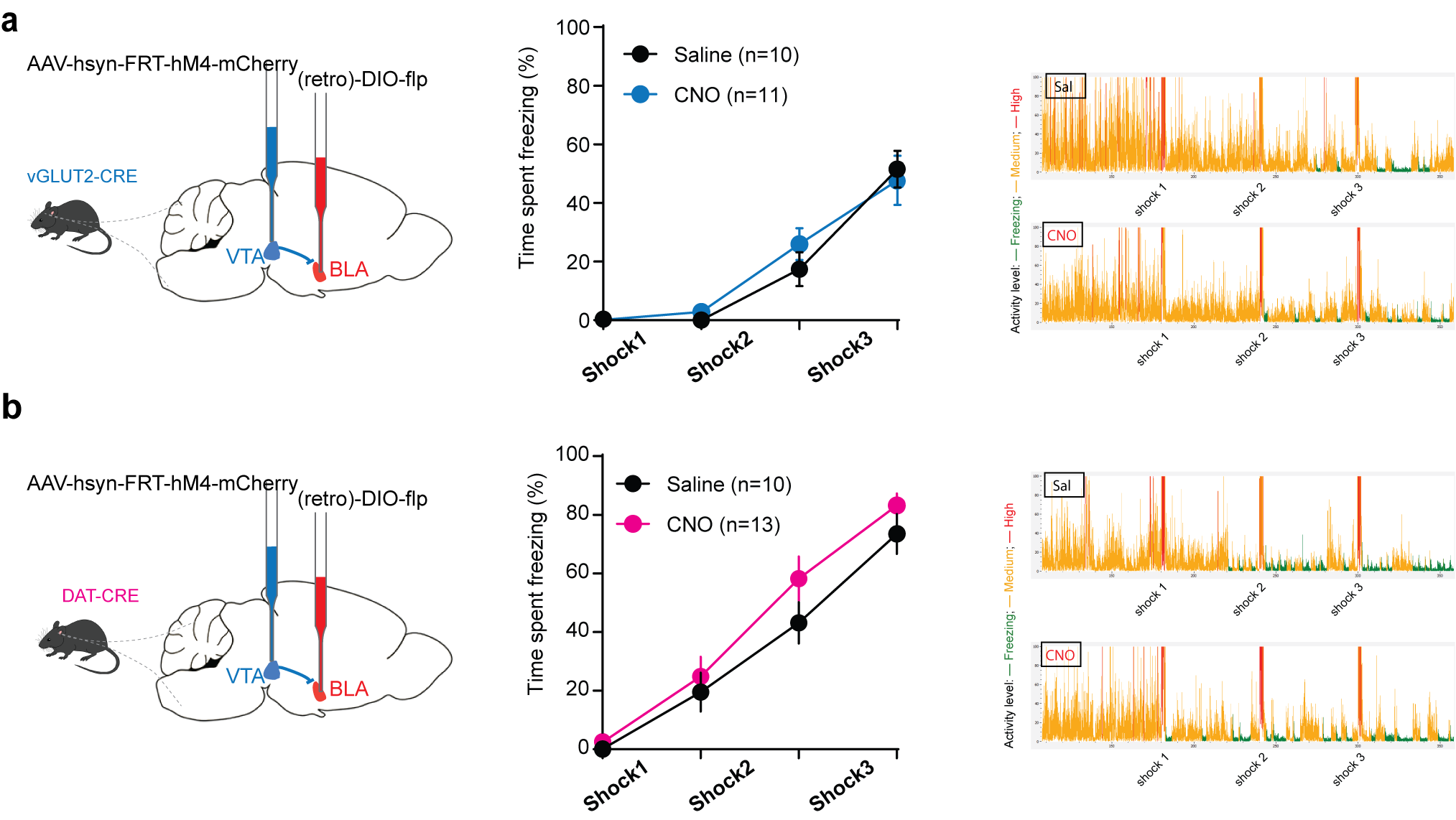

Supplement: Supplementary file 11 — Fig.S10 [file 41380_2022_1765_MOESM11_ESM.tif]

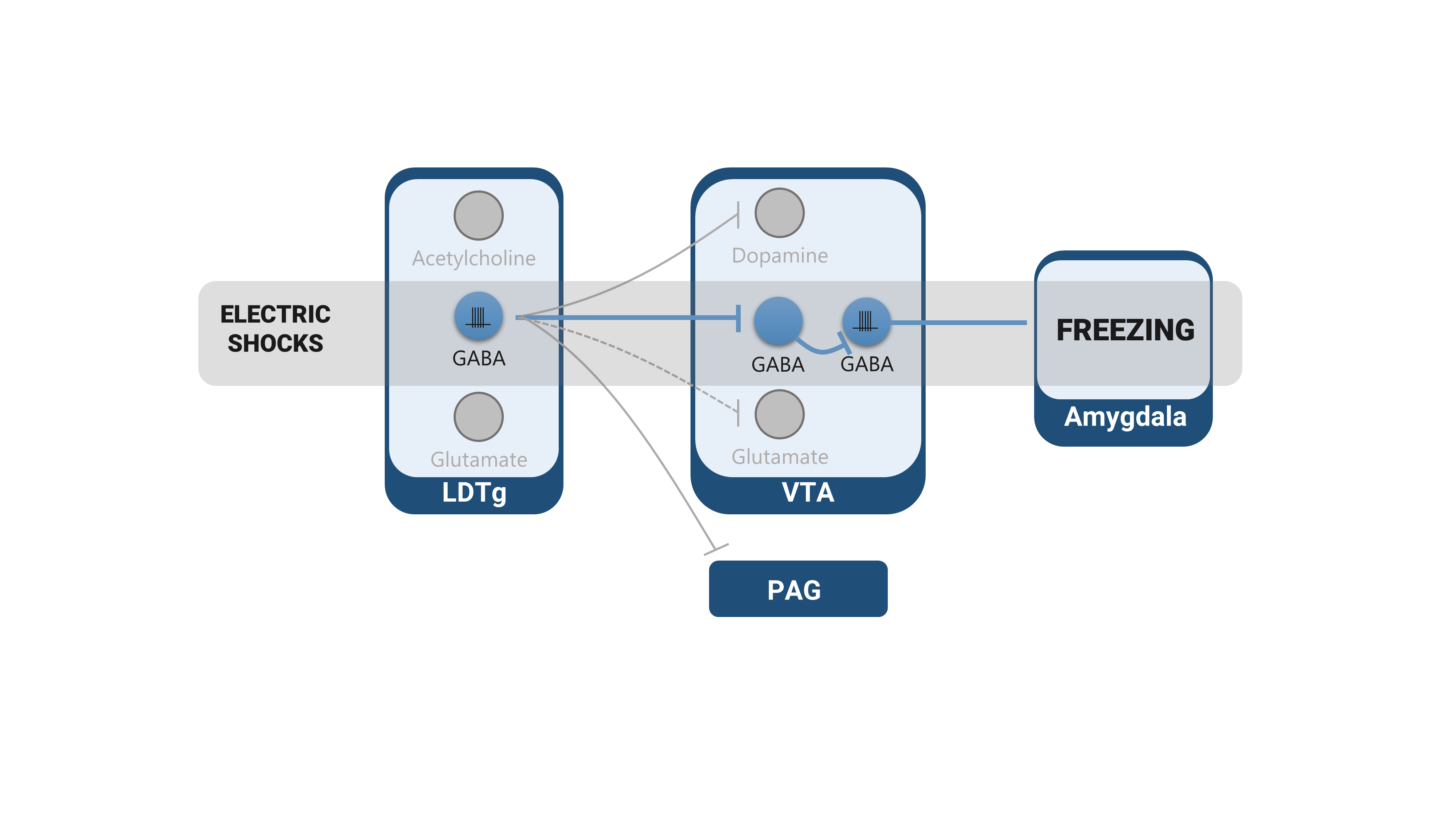

Supplement: Supplementary file 12 — Fig.S11 [file 41380_2022_1765_MOESM12_ESM.tif]
